# Cell-Nonautonomous Effects of dFOXO/DAF-16 in Aging

**DOI:** 10.1016/j.celrep.2014.01.015

**Published:** 2014-02-06

**Authors:** Nazif Alic, Jennifer M. Tullet, Teresa Niccoli, Susan Broughton, Matthew P. Hoddinott, Cathy Slack, David Gems, Linda Partridge

**Affiliations:** 1 Department of Genetics, Evolution and Environment, Institute of Healthy Ageing, University College London, Darwin Building, Gower Street, London WC1E 6BT, UK; 2 Max Planck Institute for Biology of Ageing, Joseph-Stelzmann-Strasse 9b, 50931 Cologne, Germany

## Abstract

*Drosophila melanogaster* and *Caenorhabditis elegans* each carry a single representative of the Forkhead box O (FoxO) family of transcription factors, dFOXO and DAF-16, respectively. Both are required for lifespan extension by reduced insulin/Igf signaling, and their activation in key tissues can extend lifespan. Aging of these tissues may limit lifespan. Alternatively, FoxOs may promote longevity cell nonautonomously by signaling to themselves (FoxO to FoxO) or other factors (FoxO to other) in distal tissues. Here, we show that activation of dFOXO and DAF-16 in the gut/fat body does not require *dfoxo*/*daf-16* elsewhere to extend lifespan. Rather, in *Drosophila*, activation of dFOXO in the gut/fat body or in neuroendocrine cells acts on other organs to promote healthy aging by signaling to other, as-yet-unidentified factors. Whereas FoxO-to-FoxO signaling appears to be required for metabolic homeostasis, our results pinpoint FoxO-to-other signaling as an important mechanism through which localized FoxO activity ameliorates aging.

## Highlights

• *dfoxo*/*daf-16* is not required beyond gut/adipose tissue to counteract aging
• Neuroendcrine dFOXO extends lifespan independently of *dfoxo* in other cell types
• In contrast, gut/adipose dFOXO requires *dfoxo* in other tissues to alter metabolism

## Graphical Abstract

**Figure:**
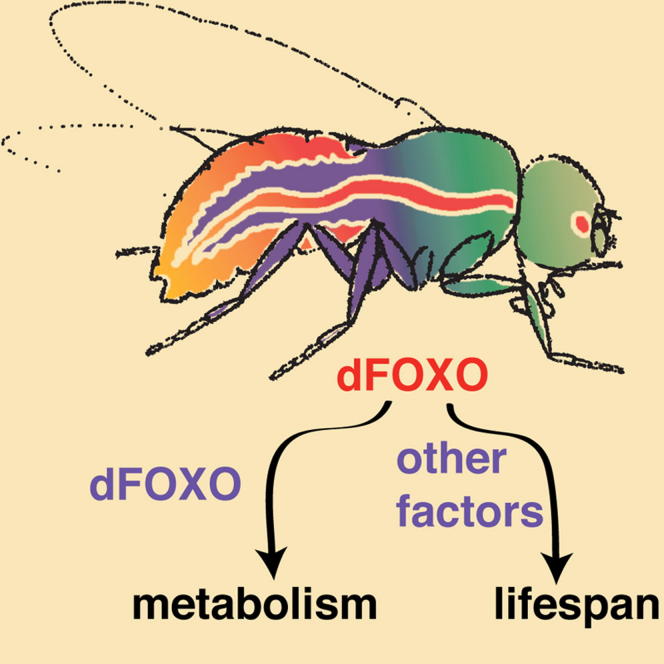

## Teaser

Activation of Forkhead box O (FoxO) transcription factors in key tissues can extend lifespan. FoxOs may promote longevity cell nonautonomously by signaling to themselves (FoxO-to-FoxO) or other factors (FoxO-to-other). The findings of Partridge and colleagues emphasize the importance of FoxO-to-other signaling in the antiaging effects of tissue-restricted induction of FoxO in the fly and worm. At the same time, the authors reveal that *Drosophila foxo* may signal to itself in distal tissues to alter fly metabolism.

## Introduction

Forkhead box O (FoxO) transcription factors (TFs) are involved in a plethora of cellular processes to regulate whole-organism physiology and are major determinants of animal lifespan (Partridge and Brüning, 2008, Salih and Brunet, 2008). Activation of FoxO-family TFs mediates the lifespan-extending effects of dampened insulin/insulin-like growth factor-like signaling (IIS) in both worms and flies (Kenyon et al., 1993, Slack et al., 2011, Yamamoto and Tatar, 2011). This evolutionary conservation appears to extend to humans, because certain genetic variants of *Foxo3A* are robustly associated with human longevity (Flachsbart et al., 2009, Kuningas et al., 2007, Willcox et al., 2008). Indeed, Forkhead-like TFs can even extend lifespan in a single-celled eukaryote, budding yeast (Postnikoff et al., 2012).

In *Drosophila melanogaster*, tissue-restricted activation of *Drosophila foxo* (*dfoxo*) is sufficient to extend lifespan (Demontis and Perrimon, 2010, Giannakou et al., 2004, Hwangbo et al., 2004). Such an increase in *dfoxo* activity confined to key tissues could promote whole-organism survival in two mutually compatible ways: cell autonomously and cell nonautonomously. The lifespan of the animal could be limited by pathology in a particular organ, so that cell-autonomous action of *dfoxo* in that organ alone could promote longevity (Rera et al., 2013). In addition, healthy aging may involve the coordinated action of multiple organ systems, with *dfoxo* in one organ altering whole-organism physiology through systemic changes (Demontis and Perrimon, 2010, Hwangbo et al., 2004, Rera et al., 2013). For example, adult-onset induction of *dfoxo* in the midgut and abdominal fat body (equivalent to mammalian liver and adipose) activates the transcription of *Drosophila insulin-like peptide* (*dilp*) *6* in the fat body, whereas in muscle *dfoxo* represses the activin ligand *dowdle*, and these endocrine signals have a distal effect on the median neurosecretory cells (mNSCs) in the brain, resulting in lowered DILP2 peptide in circulation (Bai et al., 2012, Bai et al., 2013). Importantly, upregulation of *dilp6* is required for the beneficial effect of *dfoxo* on lifespan (Bai et al., 2012). However, whether this requires *dfoxo* in tissues other than the ones producing the DILP6 signal remains unexamined.

The single *Caenorhabditis elegans* FoxO ortholog, DAF-16, can act both cell autonomously and cell nonautonomously to regulate gene expression (Libina et al., 2003, Murphy et al., 2007, Qi et al., 2012, Zhang et al., 2013). DAF-16 activity in one tissue can induce DAF-16 activity in another in a process of tissue entrainment mediated by altered expression of an insulin-like peptide (Murphy et al., 2007), which is highly reminiscent of the situation in the fly. For this reason, it has been widely believed that the fruit fly’s *dfoxo* acts from specific cells to activate dFOXO in the whole animal in an instance of *dfoxo*-to-*dfoxo* signaling (Bai et al., 2012, Bai et al., 2013, Demontis and Perrimon, 2010, Hwangbo et al., 2004). However, the relevance of this tissue entrainment for *Drosophila* lifespan has not been experimentally tested. Indeed, there is a growing awareness that FoxOs in one tissue can also signal to other factors elsewhere, i.e., FoxO-to-other signaling. In the worm, DAF-16 activity in one tissue can elicit *daf-16*-independent responses in the receiving tissues (Qi et al., 2012, Zhang et al., 2013). The existence and relevance of *dfoxo*-to-other intertissue signaling is unexplored in *Drosophila*.

Here, we establish the relevance to aging of the cell-nonautonomous effects of *dfoxo*, differentiating between *dfoxo*-to-*dfoxo* and *dfoxo*-to-other signaling in adult *Drosophila*. We find that *dfoxo*-to-*dfoxo* signaling does not affect aging and confirm that the same is true of the worm *daf-16*. On the other hand, *dfoxo* in the gut and fat body can promote health of the neuromuscular system, possibly via transcriptional regulation of a secreted neuropeptide-like molecule, and *dfoxo* in mNSCs can extend lifespan. Both effects are independent of *dfoxo*’s presence in other tissues, demonstrating the relevance of *dfoxo*-to-other signaling in *Drosophila* aging. At the same time, *dfoxo*-to-*dfoxo* signaling is required for the metabolic effects of localized *dfoxo* induction, showing that distinct physiological effects of tissue-restricted *dfoxo* activation are mediated by different signaling routes.

## Results

### *dfoxo*-to-*dfoxo* Signaling in *Drosophila* Is Dispensable for Extension of Lifespan by Gut/Fat Body or mNSC *dfoxo*

To examine whether activation of dFOXO in other tissues contributes to the lifespan-extending effects of induction of *dfoxo* in the adult gut and fat body, we generated strains where the tissue-restricted induction of *dfoxo* could be triggered by the RU486 inducer in either an otherwise wild-type or a *dfoxo*-null background (*S*_*1*_*106>dfoxo* or *dfoxoΔ*/*Δ S*_*1*_*106>dfoxo*). We used females, where the effects of *dfoxo* activation on aging are clearly observed (Giannakou et al., 2004). Because the lifespan effects of ectopic *dfoxo* expression can be conditional on the nutritional status of the animal (Bai et al., 2012, Min et al., 2008), we used a food with the optimal amount of dietary yeast (10% weight/volume) for lifespan under our laboratory conditions (Bass et al., 2007) and where expression of *dfoxo* targeted to adult gut and fat body robustly extends lifespan (Giannakou et al., 2008). Importantly, on this food, lifespan is maximized so that the effects of *dfoxo* can be studied as additional to the beneficial effects of the diet.

**Figure 1:**
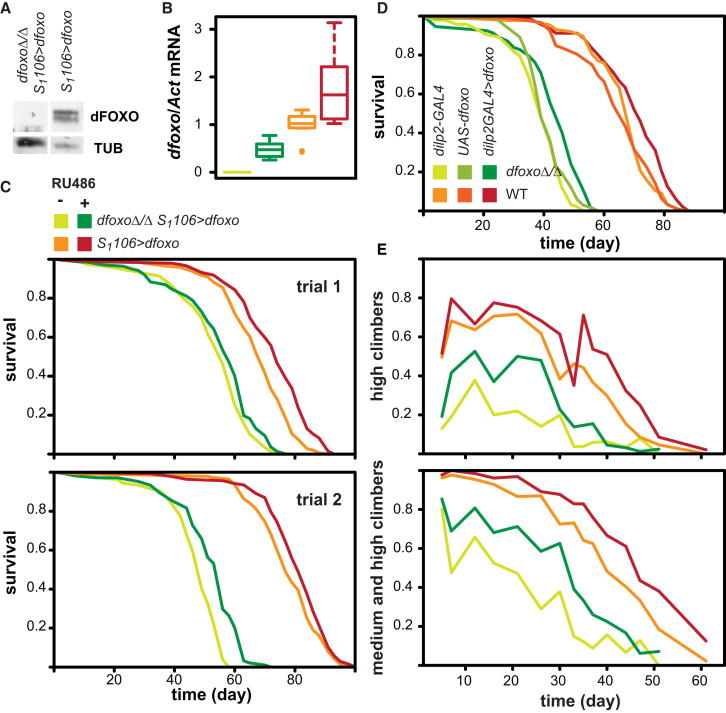
*dfoxo*-to-*dfoxo* Signaling Is Not Required for the Antiaging Effects of Increased dFOXO Activity in the Gut/Fat Body or mNSC (A) Western blots of dFOXO and the tubulin loading control on whole-fly protein extracts from *S*_*1*_*106>dfoxo* or *dfoxoΔ*/*Δ S*_*1*_*106>dfoxo* female flies in the absence of the inducer. (B) *dfoxo* transcript levels (relative to *Act* and with *S*_*1*_*106>dfoxo* -RU486 set to 1) in *S*_*1*_*106>dfoxo* or *dfoxoΔ*/*Δ S*_*1*_*106>dfoxo* female flies fed or not RU486. (C) Survival of *S*_*1*_*106>dfoxo* or *dfoxoΔ*/*Δ S*_*1*_*106>dfoxo* female flies in presence or absence of RU486 determined in two experimental trials (top and bottom panel). (D) Survival of *dilp2GAL4>dfoxo* female flies, or the two genetic controls (*dilp2-GAL4* or *UAS-dfoxo* alone), in wild-type (WT) or *dfoxoΔ*/*Δ* backgrounds. (E) The proportion of high climbers (top panel) or combined medium and high climbers (bottom panel) in three cohorts (combined) of *S*_*1*_*106>dfoxo* or *dfoxoΔ*/*Δ S*_*1*_*106>dfoxo* female flies in the presence or absence of RU486. Note the same color code is used in (B), (C), and (E) and is given in (C). See Table 1 for statistical analysis of data in (B)–(E). Where used, box plots indicate median, first and third quartile, data range, and outliers.See also Figure S1.

We found no detectable expression of dFOXO protein or of *dfoxo* transcript in the *dfoxoΔ*/*Δ S*_*1*_*106>dfoxo* females in the absence of the inducer (Figures 1A and 1B). Feeding RU486 for 5 days resulted in equivalent increases in *dfoxo* transcript in *S*_*1*_*106>dfoxo* and *dfoxoΔ*/*Δ S*_*1*_*106>dfoxo* females (Figure 1B; see Table 1 for detailed statistical analysis). The *S*_*1*_*106* driver has been thoroughly characterized and, in the female fly, only drives expression in the gut and fat body (Poirier et al., 2008). To ensure the flies are experiencing the same nutritional conditions, we examined their feeding behavior with the proboscis-extension assay (Wong et al., 2009) and found no significant differences (Figure S1A).

We examined the effect on lifespan resulting from the presence of the inducer in the *S*_*1*_*106>dfoxo* and *dfoxoΔ*/*Δ S*_*1*_*106>dfoxo* lines in two sequential, independent, experimental trials (Figure 1C), recording deaths of over 1,000 flies in total. The presence of RU486 from day 2 of adulthood extended the median lifespan of *S*_*1*_*106>dfoxo* females on average by 6% (log-rank test p < 0.05 for each trial; Figure 1C). The magnitude of the effect was less than previously reported (Giannakou et al., 2004) but is consistent with more recent work in our laboratory (Giannakou et al., 2008) and with six other independent trials performed in the course of the last 4 years (2008–2012, average median extension 5%; Figure S1B). The lifespan of *dfoxo*-null flies was also extended by a similar percentage (average 10%, log-rank test p < 0.05 for each trial; Figure 1C). Thus, the presence of *dfoxo* in the rest of the body is not required for the lifespan-extending effects of its induction in the gut/fat body.

Flies lacking *dfoxo* have short lifespans (Figure 1C), possibly due to developmental effects of the mutation (Slack et al., 2011), complicating the direct comparison between effects of RU486 in the two lines. Cox proportional hazards (CPH) is a survival analysis that allows for the significance of several covariates and their interactions to be examined. To establish whether there was any statistically significant difference in the response of *dfoxo*-null and wild-type flies to RU486, we combined the two experimental trials and analyzed the survival data using a mixed-effects Cox proportional hazards (MECPH) model (Table 1). Both RU486 (30% reduction in risk of death, p = 2 × 10^−4^) and the presence of genomic *dfoxo* (p < 10^−15^) had a significant effect on lifespan, but their interaction did not (p = 0.95). The absence of a significant interaction confirms that the effect of RU486 did not differ between the lines and hence that the presence of *dfoxo* elsewhere in the body does not affect the extension of lifespan by induction of *dfoxo* in the gut and fat body. Thus, tissue entrainment through *dfoxo*-to-*dfoxo* signaling is not required for longevity.

This result indicated that either *dfoxo* acts cell autonomously to extend lifespan or that *dfoxo* in one tissue activates *dfoxo*-independent longevity-assurance mechanisms in other tissues. The latter would occur through dFOXO-to-other signaling, as has been observed for DAF-16 (Qi et al., 2012, Zhang et al., 2013). To further test for *dfoxo*-to-other signaling, we manipulated the levels of *dfoxo* in cells whose prominent function is in adult endocrine signaling. mNSCs in the adult brain play an important role in aging by producing DILP2, DILP3, and DILP5 (Broughton et al., 2005) and possibly other endocrine signals. Expressing *dfoxo* specifically in the mNSC, using a *dilp2-GAL4* driver, extended the lifespan of female flies in both wild-type and *dfoxo* nulls (p < 0.01 to either control in both backgrounds; Figure 1D; Table 1). CPH analysis found significant effects of the genomic *dfoxo* (p < 10^−15^) and its induction in *dilp2GAL4>dfoxo* flies (50% reduction in risk of death, p = 2.2 × 10^−5^) on lifespan but no evidence for a significant interaction between them (p = 0.33; Table 1). This confirmed that the effect on lifespan is independent of *dfoxo* in tissues other than the mNSCs.

This longevity phenotype must represent a gain of function in the mNSC, because the ablation of mNSCs, representing a loss of function in these cells, requires *dfoxo* to extend lifespan (Slack et al., 2011). Indeed, we observed no significant changes in the mRNA levels of *dilp2*, *dilp3*, and *dilp5* upon induction of *dfoxo* in mNSCs (Figures S1C and S1D). Furthermore, we found no changes in the mRNA levels of any *dilp*s detectable in whole adults (*dilp2* through to *dilp7*), including *dilp6* (Figure S1D), or their binding partner and regulator, *Imp-L2* (Alic et al., 2011b) (Figure S1E), upon activation of *dfoxo* in the mNSCs, confirming that *dilp2GAL4 > dfoxo* flies are not experiencing any alterations in systemic IIS activity. Because the principal role of these cells is in endocrine signaling, the physiological effects of *dfoxo* activation in the mNSCs are most likely to be mediated by *dfoxo*-to-other signaling.

### Gut/Fat Body *dfoxo* Acts at a Distance Independently of *dfoxo* in Target Tissues

To further investigate the role of *dfoxo*-to-other signaling in fly physiology, we examined the beneficial effects of gut/fat body induction of *dfoxo* on the neuromuscular system, an organ system distal to the site of *dfoxo* activation in our model. The ability of flies to climb a vertical surface is a suitable physiological measure of the performance of this organ system and is susceptible to aging (Cook-Wiens and Grotewiel, 2002). We scored the number of low, medium, and high climbers in three cohorts of ∼15 individuals of *S*_*1*_*106>dfoxo* or *dfoxoΔ*/*Δ S*_*1*_*106>dfoxo* genotype in the presence or absence of RU486 over ∼10 weeks.

Induction of *dfoxo* expression in the gut and fat body enhanced the climbing ability of female flies throughout their lifespan, observed as an increase in the proportion of high, or combined medium and high, climbers (Figure 1E). This enhancement could be seen in both the wild-type and *dfoxo*-null backgrounds, revealing that it is independent of *dfoxo* in other tissues. Indeed, statistical analysis (mixed-effects ordinal logistic model, Table 1) confirmed that the effect of RU486 (p = 1.8 × 10^−3^) and *dfoxo* (p = 5.4 × 10^−15^) were both significant but that their interaction was not (p = 0.12). Hence, local action of *dfoxo* in the gut and fat body has a beneficial effect on the performance of a distal organ system. This could occur through systemic effects of healthy gut and fat body or through specific signaling events. In the latter case, its independence from *dfoxo* in the distal cells is again consistent with *dfoxo*-to-other signaling.

### Gut/Fat Body *dfoxo* Regulates Expression of *Nplp4*

**Figure 2:**
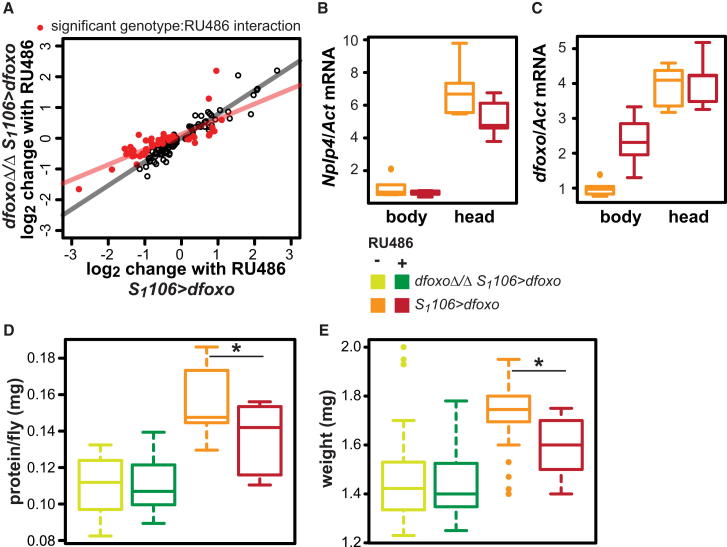
Transcriptional Regulation by Gut/Fat Body *dfoxo* and the Physiological Relevance of *dfoxo*-to-*dfoxo* Signaling (A) Log_2_ fold change in transcript levels upon RU486 administration in *S*_*1*_*106>dfoxo* (x axis) or *dfoxoΔ*/*Δ S*_*1*_*106>dfoxo* (y axis) female flies for the genes significantly changed in *S*_*1*_*106>dfoxo*. Red indicates the genes whose response to RU486 is significantly altered by genotype (significant genotype:RU486 interaction in the linear model). The red line is the regression line for these genes (slope = 0.49) and the black line is for the others (slope = 0.77). The significant difference in slope (p = 1 × 10^−7^) indicates the genes marked in red are overall less responsive to RU486 in *dfoxoΔ*/*Δ S*_*1*_*106>dfoxo* than in *S*_*1*_*106>dfoxo* female flies. Gene lists are given in Table S1. (B) *Nplp4* transcript levels (relative to *Act* and with body -RU486 set to 1) in bodies or heads of *S*_*1*_*106>dfoxo* female flies fed or not RU486. (C) *dfoxo* transcript levels (relative to *Act* and with body -RU486 set to 1) in bodies or heads of *S*_*1*_*106>dfoxo* female flies fed or not RU486. (D) Protein content of individual *S*_*1*_*106>dfoxo* or *dfoxoΔ*/*Δ S*_*1*_*106>dfoxo* female flies after 5-day feeding with RU486 or not. (E) Individual fly weight for the same conditions. Note the same color code is used in (B)–(E) and is given in (B). In (D) and (E), asterisk indicates significant difference at p < 0.05 by post hoc, pair-wise t test between − and + RU486 conditions. See Table 1 for statistical analysis of data in (B)–(E). See Figure S2 for further data.

In order to trigger *dfoxo*-to-other signaling, the gut/fat body *dfoxo* may regulate the expression of a secreted factor other than *dilp6*. To identify such a factor, we determined the whole-fly, genome-wide, transcriptional changes induced by RU486 in the *S*_*1*_*106>dfoxo* flies (Table S1; Figure 2A). We found that, besides the documented changes in *dilp6* (Bai et al., 2012), induction of the gut/fat body *dfoxo* altered the mRNA levels of another gene encoding a signal peptide targeting its protein product for secretion, *neuropeptide-like precursor 4* (*Nplp4*). The mature product of this gene is a YSY peptide of previously unknown function (Nässel and Winther, 2010). Quantitative PCR confirmed that activation of *dfoxo* led to repression of this gene (Figure 2B). Hence, *Nplp4* is a candidate for a secreted factor mediating *dfoxo*-to-other signaling. Interestingly, this gene was repressed in both heads and bodies of *S*_*1*_*106>dfoxo* females fed RU486 (p = 0.048 for RU486, p = 0.19 for body part:RU486 interaction; Figure 2B; Table 1), whereas, as expected, the induction of *dfoxo* itself was confined to the body, (p = 0.053 for body part:RU486 interaction; Figure 2C; Table 1), indicating *Nplp4* responds to dFOXO both locally and distally.

### Importance of *dfoxo*-to-*dfoxo* Signaling to *Drosophila* Metabolism

Although *dfoxo*-to-*dfoxo* signaling is not required for lifespan extension, it may be required for other physiological changes in response to the activation of dFOXO in gut and fat body. To query the existence of these other physiological effects, we examined whether there are transcriptional changes in response to RU486 in *S*_*1*_*106>dfoxo* flies that do not occur in *dfoxoΔ*/*Δ S*_*1*_*106>dfoxo* females. We reasoned that the genes and processes that respond to RU486 in *S*_*1*_*106>dfoxo* flies but fail to do so in the *dfoxoΔ*/*Δ S*_*1*_*106>dfoxo* females may be regulated through *dfoxo*-to-*dfoxo* signaling.

Among the genes regulated by RU486 in *S*_*1*_*106>dfoxo* females, we identified all those for which the RU486-induced transcriptional change was altered by mutation of *dfoxo* by finding the genes whose transcript levels show a significant interaction between the presence of genomic *dfoxo* and its induction by RU486 in the relevant linear model (Figure 2A; Table S1). The magnitude of fold-change for these genes was reduced on average in *dfoxoΔ*/*Δ S*_*1*_*106>dfoxo* compared to *S*_*1*_*106>dfoxo* females (Figure 2A), indicating they require the presence of genomic *dfoxo* for correct expression. We confirmed the significance of this effect using a linear model (p = 1 × 10^−7^; Figure 2A and the associated caption). Note that *Nplp4* was equally repressed in *dfoxoΔ*/*Δ S*_*1*_*106>dfoxo* and *S*_*1*_*106>dfoxo* females (Table S1).

Examination of the Gene Ontology categories enriched in this group of genes revealed “proteolysis” as overrepresented (p = 3.1 × 10^−7^; Table S1), hinting that protein metabolism may be regulated through a *dfoxo*-to-*dfoxo* signal. Pursuing this lead, we found that RU486 feeding triggered a small (12%) but significant reduction in total protein content of *S*_*1*_*106>dfoxo* females and that this effect was blocked by deletion of *dfoxo* (Figure 2D; p = 3.1 × 10^−3^ for RU486 by genotype interaction; Table 1). Similar significant changes were not observed in total triglyceride, total trehalose, or total glycogen content (Figure S2A). However, due to assay variability, we cannot discount possible subtle changes in these metabolites. On the other hand, total body mass followed closely the protein content (Figure 2E; Table 1).

Surprisingly, both deletion of *dfoxo* and its induction in the gut and fat body reduced total protein content and fly weight. The two manipulations may act in different ways. The small size of *dfoxo* nulls is due to the developmental effects of the mutation (Slack et al., 2011) and, together with their reduced fecundity (Slack et al., 2011) (Figure S2B), could explain the lowered body weight and protein content. On the other hand, *S*_*1*_*106>dfoxo* was induced in adulthood and had no effect on fecundity in either wild-type or *dfoxo*-null females (Figure S2B), but it had an effect on body weight and protein content. Hence, the latter two metabolic phenotypes of dFOXO activation in gut/fat body may depend on *dfoxo*-to-*dfoxo* signaling. However, because *dfoxo* was absent in all tissues throughout development, we cannot exclude the possibility that the inability of *dfoxo* nulls to respond to RU486, for these traits, is due to developmentally altered gut/fat body function. Note that the expression pattern of the proteolysis genes, which initially led us to this phenotype, cannot mechanistically explain the loss of protein in *S*_*1*_*106>dfoxo* females upon RU486 feeding, because these genes are repressed in this condition (Figure S2C), but may rather be part of a homeostatic mechanism. Nevertheless, the results strongly indicate the effects on lifespan and metabolism of tissue-restricted activation of dFOXO can be separated by their requirement for *dfoxo*-to-other or *dfoxo*-to-*dfoxo* signaling.

### *daf-16*-to-*daf-16* Signaling in *C. elegans* Is Dispensable for Extension of Lifespan by Gut *daf-16*

The worm intestine serves a functionally similar role to the gut and fat body in *Drosophila*. It is an important *daf-16* signaling center, and increased DAF-16 activity in this organ activates DAF-16 elsewhere (Libina et al., 2003, Murphy et al., 2007). However, in the context of reduced IIS resulting from mutation in *daf-2*, *daf-16* presence solely in the intestine is sufficient substantially, but not completely, to restore the *daf-2* mutant longevity in otherwise *daf-16*-deficient worms (Libina et al., 2003). This indicates that the observable *daf-16*-to-*daf-16* signaling is not essential for lifespan extension. However, the question remains whether *daf-16*-to-*daf-16* signaling contributes to the longevity promoted by DAF-16 alone.

To investigate this, we tested for a role of *daf-16*-to-*daf-16* signaling in longevity induced by *daf-16* overexpression in the intestine. Increasing DAF-16 activity alone, within otherwise wild-type worms, has two opposing effects on lifespan. DAF-16 stimulates germline hyperplasia in a cell-nonautonomous manner (Qi et al., 2012). This shortens the animal’s lifespan and masks the second, prolongevity effect of DAF-16 (Qi et al., 2012). Indeed, semiubiquitous overexpression of *daf-16* extends lifespan only if the germline cell proliferation is blocked, e.g., by administration of 5-fluoro-2′-deoxyuridine (FUdR) (Qi et al., 2012).

**Figure 3:**
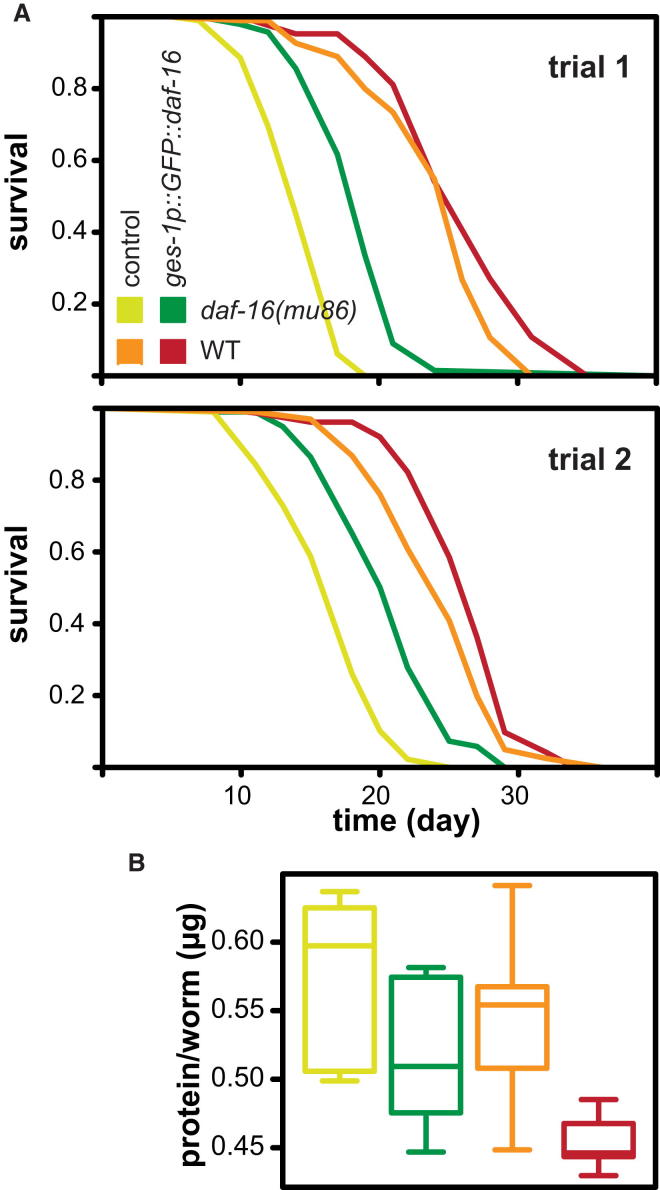
*daf-16*-to-*daf-16* Signaling Is Not Essential for the Lifespan Benefit of Increased DAF-16 Activity in the Intestine (A) Survival of wild-type (WT) and *daf-16(mu86)* worms in combination with *daf-16* overexpression from a gut-specific promoter (*muEx211[ges-1p::GFP::daf-16]*) determined in two experimental trials (top and bottom panel) in worms fed HT115 bacteria. (B) Worm protein content under the four conditions described in (A). Color codes are the same in (A) and (B). See Figure S3A for further lifespan trials and Table 1 for statistical analysis. See also Figures S3B and S3D for the effect of an independent transgene. See Figure S3C for the lifespan effects on OP50 bacteria.

Similar to Qi and coworkers, we used FUdR to reveal the lifespan extension caused by overexpression of *daf-16* from the intestinal-specific *ges-1* promoter in otherwise wild-type worms and asked whether this effect required the presence of *daf-16* in other tissues (Figure 3A). We found that intestinal activation of DAF-16 can extend lifespan of both wild-type and *daf-16*-deficient (*daf-16(mu86)*) worms fed HT115 bacteria (log-rank p < 0.05 in three out of five and three out of three assays, respectively; Figures 3A and S3A). We confirmed these findings using a second, independently derived transgene (Figure S3B).

Upon further examination, we found that the ability of the intestinal *daf-16* to extend wild-type lifespan was conditional on the food source and had a small but opposite effect when worms were fed on OP50 bacteria (Figure S3C). This is similar to the effect of gut/fat body expression of *dfoxo* on *Drosophila* lifespan, which can depend on available nutrition (Bai et al., 2012, Min et al., 2008). Importantly, however, the ability of the intestinal *daf-16* to extend lifespan in the absence of *daf-16* elsewhere was observed under all conditions, including the worms fed OP50 (Figures 3 and S3A–S3C). MECPH analysis of the combined data obtained with one of the transgenes on HT115 bacteria (Figures 3 and S3A) confirmed that both the effects of *daf-16* presence in the whole worm and its intestinal induction were significant (p < 10^−15^ for both) and revealed a significant interaction of the two main effects (p = 2 × 10^−15^; Table 1). Thus, intestinal *daf-16* extended the lifespan of the mutant more than that of the wild-type worms (Figure 3), confirming that, as in the fly (Figure 1C) and during IIS dampening in the worm (Libina et al., 2003), tissue entrainment through *daf-16*-to-*daf-16* signaling is not required for lifespan extension and, indeed, could even have the opposite effect.

Prompted by the findings in the fly (Figure 2D), we also examined if the induction of intestinal *daf-16* in worms had an effect on their total protein content. We found that the intestinal *daf-16* reduced whole-worm protein content (p < 10^−4^; Figure 3B; Table 1) on HTT15 bacteria. In contrast to the fly, we found no evidence that this reduction is prevented by the absence of *daf-16* in other tissues, and, in fact, found that mutation of *daf-16* increases the overall protein content (Figure 3B; Table 1). We obtained similar results with the second transgene (Figure S3D). Hence, this phenotype is mediated either cell autonomously by *daf-16* in the intestine or through *daf-16*-to-other signaling. Thus, whereas the physiological effect appears conserved between the fly and worm, the way it is mediated differs.

It is also of note that, similar to the lifespan effect of intestinal *daf-16* in an otherwise wild-type worm, we found this modulation of protein content conditional on the bacterial food source and neither the transgenes nor mutation of *daf-16* had any significant effect when worms were fed OP50 bacteria (data not shown). For both lifespan and protein content, the alteration of phenotype between OP50 and HTT15 is reminiscent of the lifespan effects of certain sensory mutants in *C. elegans* (Maier et al., 2010) and suggests that intestinal DAF-16 plays a role in food perception.

## Discussion

In the fly, tissue-restricted dFOXO triggers endocrine factors to cause a drop in overall, systemic, IIS activity (Bai et al., 2012, Bai et al., 2013, Demontis and Perrimon, 2010, Hwangbo et al., 2004). Because insulin signals repress the activity of FoxOs (Brunet et al., 1999), this will result in body-wide activation of dFOXO (tissue entrainment), including further activation of dFOXO in the specific tissue (positive feedback). Our results show that the tissue entrainment is not required for the beneficial effects of *dfoxo* on lifespan or on healthspan. The regulation of systemic IIS by local *dfoxo* can still be relevant to lifespan as part of a positive feedback loop. For example, the upregulation of *dilp6* by dFOXO in the fat body triggers a reduction in global IIS activity, and this, in turn, could be affecting lifespan by fine-tuning the activity of dFOXO in the fat body itself.

Under certain experimental conditions, the lifespan effects of ectopic *dfoxo* expression can be conditional on the nutrients available to the animal (Bai et al., 2012, Min et al., 2008). Hence, tissue entrainment may also have conditional relevance. In addition, our results indicate that *dfoxo*-to-*dfoxo* signaling is required for the metabolic effects of localized *dfoxo* induction, namely a drop in protein content and fly weight, and further examination may reveal roles for *dfoxo*-to-*dfoxo* signals in yet other aspects of physiology.

Both DAF-16 in *C. elegans* and dFOXO in *Drosophila* can extend lifespan from the gut/fat body without being present in other tissues. The gut and/or fat body may represent the organs most vulnerable to aging, so that DAF-16/dFOXO directly prevents the otherwise lethal age-related pathologies in these organs. This, in turn, could have indirect benefits for other organs. Indeed, there is some evidence that the health of the *Drosophila* gut limits lifespan (Rera et al., 2013). Furthermore, DAF-16/dFOXO could regulate key metabolic genes in these tissues, such as lipases, fatty acid catabolic genes, and others, effecting a shift in energy utilization toward prolonged health and survival. However, dFOXO activity in other tissues, such as the muscle (Demontis and Perrimon, 2010) or the mNSC (Figure 1D), can also extend lifespan. Although it is conceivable that multiple tissues independently and simultaneously limit lifespan, in at least some of these interventions, the relevant effects must be cell nonautonomous.

DAF-16 in one tissue is known to trigger DAF-16-independent responses in other tissues (Qi et al., 2012, Zhang et al., 2013). In one case, this is mediated by induction of a transcriptional mediator, *mdt-15*, and is required in part for the beneficial effects of the intestinal activation of DAF-16 by *daf-2(−)* on whole-organism aging (Zhang et al., 2013). Our results indicate that dFOXO may also initiate *dfoxo*-independent processes in the receiving tissues that counteract whole-organism aging. This is the most likely mechanism whereby its activity in the *Drosophila* mNSC can extend lifespan, and a similar mechanism may underlie the health benefits observed when it is induced in the gut and fat body. The search for the factors that mediate this effect of dFOXO at a distance is now of interest, and we identified *Nplp4* as a candidate. The evolutionary persistence of this FoxO-to-other signaling between the fly and the worm strongly suggests that its relevance may extend to mammals.

## Experimental Procedures

### Fly Husbandry and Experiments

All transgenes and the *dfoxo* mutant were backcrossed at least six times into the wild-type outbred Dahomey population carrying the *w*^*1118*^ mutation and cured of *Wolbachia* infection and frequently outcrossed back into the wild-type population. The Dahomey stock was collected in 1970 in Dahomey (now Benin) and has been kept in population cages maintaining its lifespan and fecundity at levels similar to freshly caught stocks. The lines were maintained, and all experiments performed, at 25°C with 60% humidity and 12 hr:12 hr light:dark cycle on sugar-yeast-agar (1SYA) food (Bass et al., 2007). Experimental flies developed at standardized densities and once-mated females were sorted on day 2 of adulthood onto food containing 200 μM RU486 (Sigma) or control food as required (15 per vial for climbing assays, five for feeding, and ten for all others). Flies were harvested on day 7 for weight and metabolite measurements and protein and RNA analysis. Sample preparation and hybridizations to Dros2 Affymetrix arrays were performed and data analyzed with LIMMA, essentially as described elsewhere (Alic et al., 2011a). For further details, see Supplemental Experimental Procedures. Gene lists are given in Table S1.

### Worm Husbandry and Experiments

Worms were maintained at 20°C unless otherwise indicated. Prior to experiments, animals were maintained at the permissive temperature and grown for at least one generation in the presence of food to assure full viability. Lifespan assays were performed on HT115 bacteria carrying empty pL4440 vector, or OP50 bacteria, in the presence of 10 μM FUdR. Worms were placed on these plates at the L4 stage and scored as dead or alive every 2–3 days. For further details, see Supplemental Experimental Procedures.

### Statistical Analysis

Analyses were performed in JMP (SAS) or R. Further details are given in Table 1 and Supplemental Experimental Procedures. To determine difference in slopes of the regression lines between the two gene sets in Figure 2A, the linear model was fitted with RU486-induced response in *dfoxoΔ*/*Δ S*_*1*_*106>dfoxo* as the dependent variable and the response in *S*_*1*_*106>dfoxo* (continuous) and gene set (categorical) as explanatory variables, testing for the significance of the interaction term.

## Figures and Tables

**Table 1:** Statistical Analysis

Relevant Figure	Model and Description	Random Effect	Coefficient^a^	Estimate^b^	SE	p Value
Figure 1**B**	**mixed-effects linear**					

	n = 7–8	batch	intercept	0.81	0.15	0.11
			*dfoxo*	0.571	7.1 × 10^−2^	<10^−4^
			RU486	0.311	7.1 × 10^−2^	2.0 × 10^−4^
			*dfoxo*:RU486	6.6 × 10^−2^	7.1 × 10^−2^	0.36

Figure 1**C**	**MECPH**					

	1,050 deaths (1,078 total)	experimental trial	*dfoxo*	−2.1	0.11	<10^−15^
			RU486	−0.34	9.1 × 10^−2^	2 × 10^−4^
			*dfoxo*:RU486	8.5 × 10^−3^	0.13	0.95

Figure 1**D**	**CPH**					

	533 deaths (545 total)	NA	*dfoxo*	−3.26	0.22	<10^−15^
			*UAS-dfoxo*	−0.23	0.15	0.13
			*dilp2GAL4>dfoxo*	−0.65	0.15	2.2 × 10^−5^
			*dfoxo*:*UAS-dfoxo*	0.28	0.21	0.19
			*dfoxo*:*dilp2GAL4>dfoxo*	0.21	0.22	0.33

Figure 1**E**	**mixed-effects ordinal logistic**					

	total observations = 2,179	biological repeat (vial)	time	−7.1 × 10^−2^	7.8 × 10^−3^	<10^−15^
			*dfoxo*	2.1	0.27	5.4 × 10^−15^
			RU486	0.81	0.26	1.8 × 10^−3^
			time:*dfoxo*	−5.4 × 10^−3^	9.8 × 10^−3^	0.58
			time:RU486	2.9 × 10^−3^	0.01	0.78
			*dfoxo*:RU486	−0.59	0.38	0.12
			time:*dfoxo*:RU486	8.1 × 10^−3^	1.3 × 10^−2^	0.54

Figure 2**B**	**linear**					

	n = 5	NA	intercept	3.4	0.25	<10^−4^
			head	2.6	0.25	<10^−4^
			RU486	−0.54	0.25	4.8 × 10^−2^
			head:RU486	−0.34	0.25	0.19

Figure 2**C**	**linear**					

	n = 5	NA	intercept	2.8	0.25	<10^−4^
			head	1.2	0.25	<10^−4^
			RU486	0.38	0.25	1.8 × 10^−2^
			head:RU486	−0.28	0.25	5.4 × 10^−2^

Figure 2**D**	**mixed-effects linear**					

	n = 8–10	batch	intercept	0.12	1.4 × 10^−2^	6.8 × 10^−2^
			*dfoxo*	1.7 × 10^−2^	1.8 × 10^−3^	<10^−4^
			RU486	−5.5 × 10^−3^	1.8 × 10^−3^	4 × 10^−3^
			*dfoxo*:RU486	−5.7 × 10^−3^	1.8 × 10^−3^	3.1 × 10^−3^

Figure 2**E**	**mixed-effects linear**					

	n = 27–30	batch	intercept	1.6	0.03	<10^−4^
			*dfoxo*	9.6 × 10^−2^	1.3 × 10^−2^	<10^−4^
			RU486	−3.8 × 10^−2^	1.3 × 10^−2^	3.3 × 10^−3^
			*dfoxo*:RU486	−2.6 × 10^−2^	1.3 × 10^−2^	0.04

Figures 3**A and**S3**A**	**MECPH**					

	944 deaths (1,128 total)	experimental trial	*daf-16*	−2.5	0.11	<10^−15^
			intestinal *daf-16*	−1.3	0.10	<10^−15^
			*daf-16*:intestinal *daf-16*	1.1	0.14	2 × 10^−15^

Figure 3**B**	**linear**					

	n = 8–16	NA	intercept	0.52	7.9 × 10^−3^	<10^−4^
			*daf-16*	−2.3 × 10^−2^	7.9 × 10^−3^	<10^−4^
			intestinal *daf-16*	−3.7 × 10^−2^	7.9 × 10^−3^	6.2 × 10^−3^
			*daf-16*:intestinal *daf-16*	−9.3 × 10^−3^	7.9 × 10^−3^	0.24

a In all models, the effect of presence of *dfoxo* (*daf-16*) or its induction (overexpression) is examined; *dilp2-GAL4* was used as reference for *dilp2GAL4>dfoxo* and *UAS-dfoxo*; “body” was used as reference for body versus head comparisons; “:” indicates interaction term.

b For mixed-effect linear models and linear models, the coefficient estimates either have no units because they are derived from the *dfoxo*/*Act* or *Nplp4*/*Act* transcript ratios (for Figures 1B, 2B, and 2C) or are given in mg (for Figures 2D and 2E) or μg (Figure 3B). For MECPH and CPH models, the coefficient estimate is the natural log of the hazard ratio, where a negative value indicates a beneficial effect on survival. For mixed-effect ordinal logistic, these are log-transformed odds of climbing high, where a negative value indicates a reduction in climbing ability.

## Accession Numbers

The ArrayExpress accession number for the array data reported in this paper is E-MTAB-1232.

## Supplemental Information

Document S1. Supplemental Experimental Procedures and Figures S1–S3

Table S1. Supplemental Data

Document S2. Article plus Supplemental Information

## References

[bib1] Alic N., Andrews T.D., Giannakou M.E., Papatheodorou I., Slack C., Hoddinott M.P., Cochemé H.M., Schuster E.F., Thornton J.M., Partridge L. (2011). Genome-wide dFOXO targets and topology of the transcriptomic response to stress and insulin signalling. Mol. Syst. Biol..

[bib2] Alic N., Hoddinott M.P., Vinti G., Partridge L. (2011). Lifespan extension by increased expression of the Drosophila homologue of the IGFBP7 tumour suppressor. Aging Cell.

[bib3] Bai H., Kang P., Tatar M. (2012). Drosophila insulin-like peptide-6 (dilp6) expression from fat body extends lifespan and represses secretion of Drosophila insulin-like peptide-2 from the brain. Aging Cell.

[bib4] Bai H., Kang P., Hernandez A.M., Tatar M. (2013). Activin signaling targeted by insulin/dFOXO regulates aging and muscle proteostasis in Drosophila. PLoS Genet..

[bib5] Bass T.M., Grandison R.C., Wong R., Martinez P., Partridge L., Piper M.D. (2007). Optimization of dietary restriction protocols in Drosophila. J. Gerontol. A Biol. Sci. Med. Sci..

[bib6] Broughton S.J., Piper M.D., Ikeya T., Bass T.M., Jacobson J., Driege Y., Martinez P., Hafen E., Withers D.J., Leevers S.J., Partridge L. (2005). Longer lifespan, altered metabolism, and stress resistance in Drosophila from ablation of cells making insulin-like ligands. Proc. Natl. Acad. Sci. USA.

[bib7] Brunet A., Bonni A., Zigmond M.J., Lin M.Z., Juo P., Hu L.S., Anderson M.J., Arden K.C., Blenis J., Greenberg M.E. (1999). Akt promotes cell survival by phosphorylating and inhibiting a Forkhead transcription factor. Cell.

[bib8] Cook-Wiens E., Grotewiel M.S. (2002). Dissociation between functional senescence and oxidative stress resistance in Drosophila. Exp. Gerontol..

[bib9] Demontis F., Perrimon N. (2010). FOXO/4E-BP signaling in Drosophila muscles regulates organism-wide proteostasis during aging. Cell.

[bib10] Flachsbart F., Caliebe A., Kleindorp R., Blanché H., von Eller-Eberstein H., Nikolaus S., Schreiber S., Nebel A. (2009). Association of FOXO3A variation with human longevity confirmed in German centenarians. Proc. Natl. Acad. Sci. USA.

[bib11] Giannakou M.E., Goss M., Jünger M.A., Hafen E., Leevers S.J., Partridge L. (2004). Long-lived Drosophila with overexpressed dFOXO in adult fat body. Science.

[bib12] Giannakou M.E., Goss M., Partridge L. (2008). Role of dFOXO in lifespan extension by dietary restriction in Drosophila melanogaster: not required, but its activity modulates the response. Aging Cell.

[bib13] Hwangbo D.S., Gershman B., Tu M.P., Palmer M., Tatar M. (2004). Drosophila dFOXO controls lifespan and regulates insulin signalling in brain and fat body. Nature.

[bib14] Kenyon C., Chang J., Gensch E., Rudner A., Tabtiang R. (1993). A C. elegans mutant that lives twice as long as wild type. Nature.

[bib15] Kuningas M., Mägi R., Westendorp R.G., Slagboom P.E., Remm M., van Heemst D. (2007). Haplotypes in the human Foxo1a and Foxo3a genes; impact on disease and mortality at old age. Eur. J. Hum. Genet..

[bib16] Libina N., Berman J.R., Kenyon C. (2003). Tissue-specific activities of C. elegans DAF-16 in the regulation of lifespan. Cell.

[bib17] Maier W., Adilov B., Regenass M., Alcedo J. (2010). A neuromedin U receptor acts with the sensory system to modulate food type-dependent effects on C. elegans lifespan. PLoS Biol..

[bib18] Min K.J., Yamamoto R., Buch S., Pankratz M., Tatar M. (2008). Drosophila lifespan control by dietary restriction independent of insulin-like signaling. Aging Cell.

[bib19] Murphy C.T., Lee S.J., Kenyon C. (2007). Tissue entrainment by feedback regulation of insulin gene expression in the endoderm of Caenorhabditis elegans. Proc. Natl. Acad. Sci. USA.

[bib20] Nässel D.R., Winther A.M. (2010). Drosophila neuropeptides in regulation of physiology and behavior. Prog. Neurobiol..

[bib21] Partridge L., Brüning J.C. (2008). Forkhead transcription factors and ageing. Oncogene.

[bib22] Poirier L., Shane A., Zheng J., Seroude L. (2008). Characterization of the Drosophila gene-switch system in aging studies: a cautionary tale. Aging Cell.

[bib23] Postnikoff S.D., Malo M.E., Wong B., Harkness T.A. (2012). The yeast forkhead transcription factors fkh1 and fkh2 regulate lifespan and stress response together with the anaphase-promoting complex. PLoS Genet..

[bib24] Qi W., Huang X., Neumann-Haefelin E., Schulze E., Baumeister R. (2012). Cell-nonautonomous signaling of FOXO/DAF-16 to the stem cells of Caenorhabditis elegans. PLoS Genet..

[bib25] Rera M., Azizi M.J., Walker D.W. (2013). Organ-specific mediation of lifespan extension: more than a gut feeling?. Ageing Res. Rev..

[bib26] Salih D.A., Brunet A. (2008). FoxO transcription factors in the maintenance of cellular homeostasis during aging. Curr. Opin. Cell Biol..

[bib27] Slack C., Giannakou M.E., Foley A., Goss M., Partridge L. (2011). dFOXO-independent effects of reduced insulin-like signaling in Drosophila. Aging Cell.

[bib28] Willcox B.J., Donlon T.A., He Q., Chen R., Grove J.S., Yano K., Masaki K.H., Willcox D.C., Rodriguez B., Curb J.D. (2008). FOXO3A genotype is strongly associated with human longevity. Proc. Natl. Acad. Sci. USA.

[bib29] Wong R., Piper M.D., Wertheim B., Partridge L. (2009). Quantification of food intake in Drosophila. PLoS ONE.

[bib30] Yamamoto R., Tatar M. (2011). Insulin receptor substrate chico acts with the transcription factor FOXO to extend Drosophila lifespan. Aging Cell.

[bib31] Zhang P., Judy M., Lee S.J., Kenyon C. (2013). Direct and indirect gene regulation by a life-extending FOXO protein in C. elegans: roles for GATA factors and lipid gene regulators. Cell Metab..

