# Supplementary material for: Cell-Nonautonomous Effects of dFOXO/DAF-16 in Aging
Source: Cell Rep. 2014 Feb 6;6(4):608–16. doi: 10.1016/j.celrep.2014.01.015 (PMC3969275; doi:10.1016/j.celrep.2014.01.015)
Supplement: Document S1. Supplemental Experimental Procedures and Figures S1–S3 [file mmc1.pdf]

Cell Reports

Supplemental Information

Cell-Nonautonomous Effects of dFOXO/DAF-16 in Aging

Nazif Alic, Jennifer M. Tullet, Teresa Niccoli, Susan Broughton, Matthew P. Hoddinott, Cathy Slack,

David Gems, and Linda Partridge

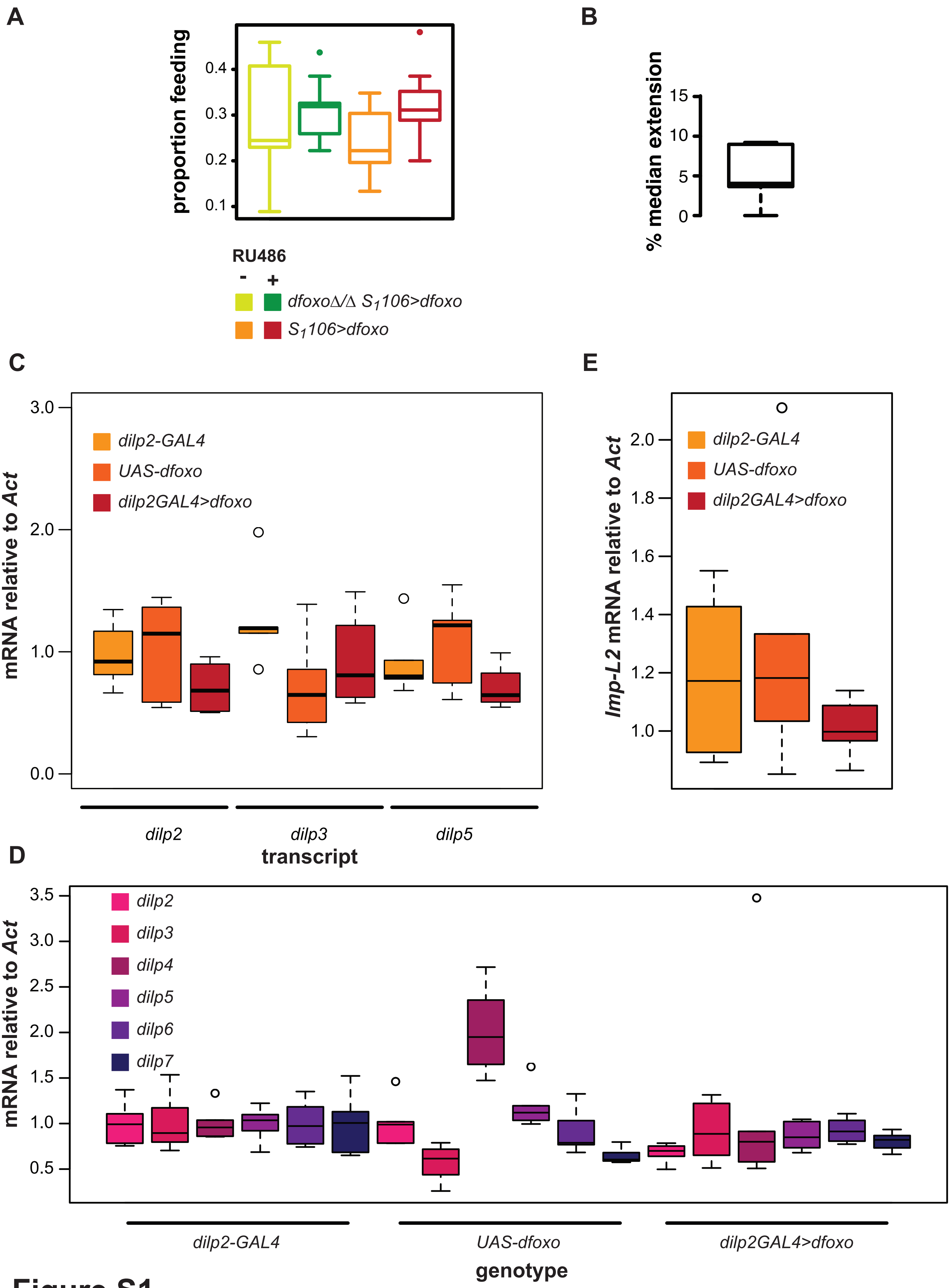
A
proportion feeding
0.4
0.3
0.2
0.1
RU486
-
+
dfoxoΔ/Δ S1106>dfoxo
S1106>dfoxo
B
% median extension
15
10
5
0
C
3.0
2.0
1.0
0.0
mRNA relative to Act
dilp2-GAL4
UAS-dfoxo
dilp2GAL4>dfoxo
dilp2
dilp3
dilp5
transcript
E
Imp-L2 mRNA relative to Act
2.0
1.8
1.6
1.4
1.2
1.0
dilp2-GAL4
UAS-dfoxo
dilp2GAL4>dfoxo
D
mRNA relative to Act
3.5
3.0
2.5
2.0
1.5
1.0
0.5
dilp2
dilp3
dilp4
dilp5
dilp6
dilp7
dilp2-GAL4
UAS-dfoxo
dilp2GAL4>dfoxo
genotype

Figure S1

Figure S1

A Feeding events as proportion of total observed events (fly occurrences) per vial for the *$S_1$106>dfoxo* and *dfoxoΔ/Δ $S_1$106>dfoxo* females (15 and 9 vials per food condition, respectively) on food with or without RU486. The data were analysed with a Generalised Linear Model with binomial distribution and an overdispersion parameter. There was no significant effect of genotype (p=0.4), presence of RU486 (p=0.08) or their interaction (p=0.4). **B** Box plot representing the median lifespan extension (as % of control) caused by RU486 feeding in *$S_1$106>dfoxo* females in six independent trials. Log-rank test revealed that RU486 had a significant effect in each case ($p<0.05$). **C** mRNA levels of *dilp2*, *dilp3* and *dilp5* in heads of female flies of the indicated genotype. Data were analysed with a Linear Model and no significant effect of transcript or genotype, or any interaction, was found ($p>0.05$, n=4-5). **D** mRNA levels of *dilp2* through to *dilp7* in whole female flies of the indicated genotype, with the level in *dilp2GAL4* control set to 1 and n=6. Data were analysed with a Linear Model and no significant effect of genotype was found (p=0.2), while there was a significant effect of the transcript ($p<10^{-4}$) and a significant interaction of the two ($p=2x10^{-4}$), but in no case was *dilp2GAL4>dfoxo* significantly different to both of the controls by t-test ($p>0.05$). Note the levels of *dilp4* in the *UAS-dfoxo* control are different to *dilp4* levels in the two other genotypes, and this is most likely due to the effect of the driver alone on *dilp4* levels. *dilp1* transcript is undetectable in whole adult females. **E** mRNA levels of *Imp-L2* in whole female flies of the indicated genotype, with the level in *dilp2GAL4* control set to 1 and n=6. ANOVA revealed no significant differences (p=0.3).

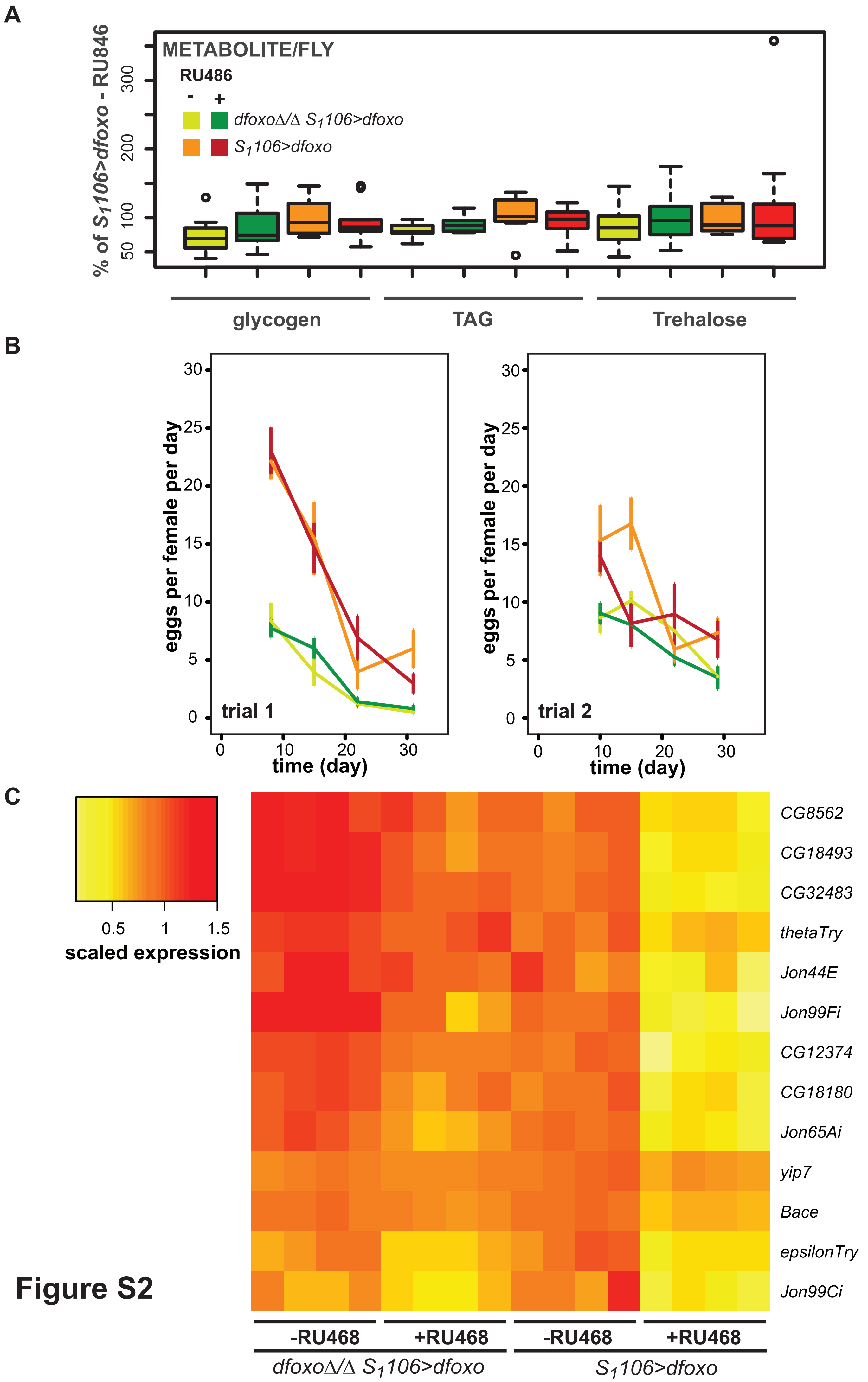
A
METABOLITE/FLY
RU486
-
+
dfoxoΔ/Δ S1106>dfoxo
S1106>dfoxo
% of S1106>dfoxo - RU846
50
100
200
300
glycogen
TAG
Trehalose
B
eggs per female per day
0
5
10
15
20
25
30
trial 1
trial 2
time (day)
0
10
20
30
C
0.5
1
1.5
scaled expression
CG8562
CG18493
CG32483
thetaTry
Jon44E
Jon99Fi
CG12374
CG18180
Jon65Ai
yip7
Bace
epsilonTry
Jon99Ci
-RU468
+RU468
-RU468
+RU468
dfoxoΔ/Δ S1106>dfoxo
S1106>dfoxo

Figure S2

Figure S2

A Trehalose, TAG and glycogen were quantified per fly for the four conditions shown (n=8-15 per condition). Data were analysed per compound with a Mixed Effect Linear Model with genotype and RU486 as main effects, their interaction, and experimental batch as random effect. Only the effect of genotype for glycogen was borderline significant (p=0.050), all others were not. **B** Eggs laid per female per 24h for the same four conditions over the first ~4 weeks of life determined in two experimental trials (two panels, means ± SEM). The data were analysed with Mixed Effect Linear Model with time, genotype and RU486 as main effects, their interactions, and experimental trial as random effect. The effects of genotype (genomic *dfoxo*), time and their interaction were all significant ($p<10^{-4}$) but RU486 or any of its interactions were not (p>0.05). The same colour code is used in A and B. **C** Heatmap of the expression levels detected by microarray analysis for proteolysis genes that show a different response to RU486 in S_1*106>dfoxo* and *dfoxo*Δ/Δ S_1*106>dfoxo* females. Each column represents a separate array with the expression levels scaled to the average of the S_1*106>dfoxo* -RU486 condition.

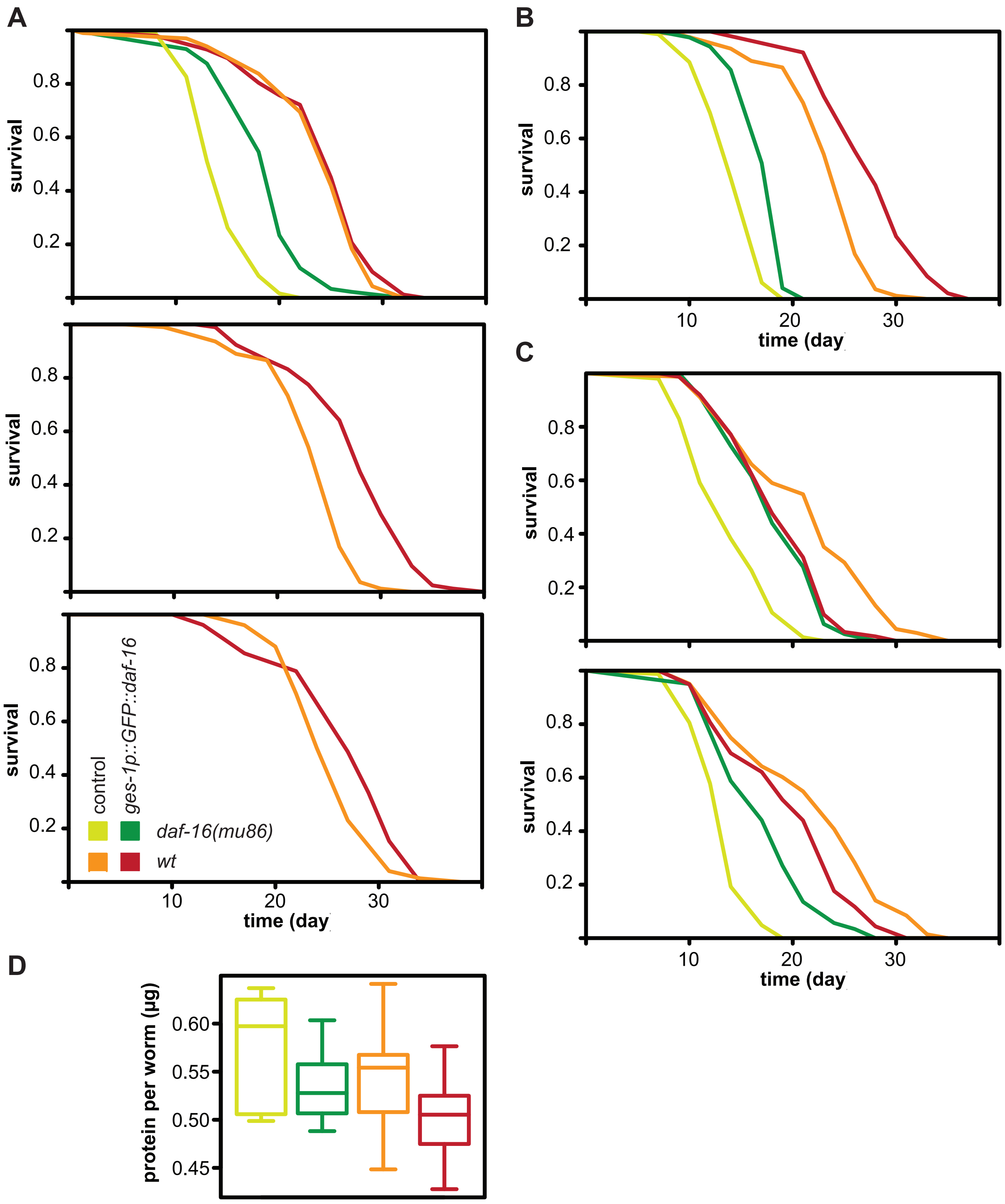
A
B
C
D
survival
0.8
0.6
0.4
0.2
10
20
30
time (day
control
ges-1p::GFP::daf-16
daf-16(mu86)
wt
protein per worm (µg)
0.60
0.55
0.50
0.45

Figure S3

Figure S3

A Further experimental trials looking at the effect of gut over-expression of *daf-16* (*muEx211[ges-1p::GFP::daf-16]*) in wild-type and *daf-16* deficient worms on HT115 bacteria. Each panel is a separate experimental trial. **B** The effect of an independently derived transgene (*muEx227[ges-1p::GFP::daf-16]*) on lifespan in wild-type and *daf-16* deficient worms on HT115 bacteria. Log-rank test detected significant differences in both wild-type and *daf-16* deficient worms ($p<10^{-4}$). **C** Two experimental trials looking at the effect of gut over-expression of *daf-16* (*muEx211[ges-1p::GFP::daf-16]*) in wild-type and *daf-16* deficient worms on OP50 bacteria. Log-rank test detected significant differences in both wild-type and *daf-16* deficient worms ($p<0.01$ and $p<10^{-4}$, respectively) in both trials. MECPH model revealed significant interaction between genomic *daf-16* and its intestinal induction ($p<10^{-15}$). **D** Protein content of worms with *daf-16* induced in the intestine (*muEx227[ges-1p::GFP::daf-16]*) in otherwise daf-16(+) or *daf-16(mu86)* worms. The data were analysed with a Linear model and the effect of transgene found significant (p=0.012), the effect of daf-16 mutation borderline significant (p=0.069) and no significant interaction (p=0.86, n=8-16). The colour code is the same in all panels and is given in A.

Table S1 Supplemental Data

Separate Excel File containing:

- List of genes differentially expressed (10% FRD) in S106>dfoxo females with RU486 treatment, with indication of the ones that show significant interaction between genotype (S106>dfoxo or dfoxo null S106>dfoxo) and RU486 (10% FDR).
- Biological process categories over-represented in genes with significant interaction term in list above.

Extended Experimental Procedures

Fly husbandry, lifespan, feeding and climbing assays

$S_1$106 (Giannakou et al., 2004; Poirier et al., 2008), *UAS-dfoxo* (Giannakou et al., 2004), *dilp2-GAL4* (Ikeya et al., 2002) and *dfoxo*Δ^{94} (Slack et al., 2011) were backcrossed at least 6 times into the wild-type outbred Dahomey population carrying the w^{1118} mutation and cured of *Wolbachia* infection, and frequently outcrossed back into the wild-type population. The Dahomey stock was collected in 1970 in Dahomey (now Benin) and has been kept in population cages maintaining its lifespan and fecundity at levels similar to freshly caught stocks. Combinations of transgenes/mutants were created using standard fly genetic techniques while avoiding population bottlenecks. *dfoxo*Δ^{94} allele was tracked with PCR (Slack et al., 2011). The lines were maintained, and all experiments performed, at 25ºC with 60% humidity and 12h:12h light:dark cycle, on sugar-yeast-agar (1SYA) food (Bass et al., 2007). Experimental flies developed at standardised densities and once-mated females were sorted on day two of adulthood onto food containing 200 μM RU486 (Sigma) or control food as required (15 per vial for climbing assays, 5 for feeding, 10 for all others). Note that the food from the exact same cook was used for the - and + RU486. Lifespan measurements were performed essentially as described (Giannakou et al., 2004). Climbing ability was scored once a week. Flies were placed in a plastic pipette (15 flies per pipette, 3 pipettes per condition), tapped to the bottom and allowed to climb for 45 seconds. Their position was then scored as low (climbing below 2 cm), medium (above 2 and below 20) and high (above 20 cm). This scoring was repeated 3 times for each pipette and averaged to the nearest fly. Feeding

observations were performed essentially as described (Wong et al., 2009) for one hour on days 7 and 8 by two observers, blinded to the experimental conditions, with flies feeding (proboscis extended to the food) or not scored multiple times for each vial. The observations were summed per vial before analysis. Flies were harvested on day seven for weight and metabolite measurement, protein and RNA analysis, by freezing in liquid N_2.

Metabolite and weight measurements

Whole fly trehalose was determined as described (Alic et al., 2011b). For triacylglycerol (TAG) and protein measurements, a single female was homogenised in 400 μl of 0.05% Tween-20 and TAG measured with Infinity TAG Reagent (Thermo Scientific), protein with BCA Protein Assay kit (Pierce) against a suitable standard. For glycogen measurements, a single female was homogenised into 100 μl of PBS 0.1% TritonX-100, and the glucose released by incubation with amyloglucosidase in the samples and glycogen standards determined with Infinity Glucose Reagent (Thermo Scientific). Flies were weighted singly or in pairs, and the two highest/lowest measurements for each condition discarded.

Western blots, qPCR and microarray analysis

Proteins were extracted and western blots performed as described (Alic et al., 2011b). RNA extractions and qPCR were performed essentially as described (Alic et al., 2011a). *dfoxo* primers used were: GTTGGCCCCAGGAGACTC and GATGAGATCCGCATAGGATAG; *Actin5C*: CACACCAAATCTTACAAAATGTGTGA and AATCCGGCCTTGCACATG; *Nplp4*: CCGGACCATGCATTGCAATG and GCGCATATCCGTAATCCGT. *dfoxo* mRNA was deemed detectable when the qPCR signal was above that

observed in the absence of reverse transcriptase. mRNA levels of *dilp2* through to 7 and *Imp-L2* were quantified as described (Alic et al., 2011b; Broughton et al., 2005; Gronke et al., 2010). Sample preparation and hybridisations to Dros2 Affymetrix arrays were performed as described (Alic et al., 2011a). The microarray data were analysed in R. They were summarised and normalised using RMA, differential expression was assessed using Linear Models and the empirical Bayes moderated t-statistic implemented in LIMMA (Bolstad et al., 2003; Irizarry et al., 2003a; Irizarry et al., 2003b) and FDR was controlled using the described procedure (Benjamini and Hochberg, 1995). Genes differentially expressed on RU486 feeding in *$S_1$106>dfoxo* females were determined (at FDR=10%), and amongst these genes the ones with significant interaction between genotype and RU486 (FDR=10%) were determined by a Linear Model (full factorial design) fitted to all the data (the four conditions). Gene Ontology Enrichment was determined with David EASE (Dennis et al., 2003). Gene lists are given in **Supplemental Data**. Raw microarray data are available from ArrayExpress under accession code: E-MTAB-1232.

Worm husbandry, lifespan and protein assays

Worms were maintained at 20°C unless otherwise indicated. The following strains were used: N2s derived from the Caenorhabditis Genetics Center male N2 line, XA2954 *daf-16(mu86)* derived from CF1038 *daf-16(mu86)* (Lin et al., 2001), GA1064 *muEx211[ges-1p::GFP::daf-16]*, GA1065 *daf-16(mu86) muEx211[ges-1p::GFP::daf-16]*, GA1067 *muEx227[ges-1p::GFP::daf-16]*, GA1066 *daf-16(mu86) muEx227[ges-1p::GFP::daf-16]*. The latter four strains were created by crossing CF1514 *daf-16(mu86); daf-*

2(e1370) muEx211[ges-1p::GFP::daf-16] or CF1595 *daf-16(mu86); daf-2(e1370) muEx227[ges-1p::GFP::daf-16]* (Libina et al., 2003) with the N2 strain and isolating wild type and *daf-16* mutant worms carrying the extrachromosomal array using three-primer PCR: F: CGGTGACCATCTAGAGTCACA; In: CCAATAGCTGGAGAAACACGA and; R: CTAGGAGGAAAAGCCATTTGT (Love et al., 2010), without further outcrossing. Prior to experiments animals were maintained at the permissive temperature and grown for at least one generation in the presence of food to assure full viability. Lifespan assays were performed on HT115 bacteria carrying empty pL4440 vector, or OP50 bacteria, in the presence of 10μM FUdR. Worms were placed on these plates at the L4 stage and scored as dead or alive every 2-3 days. For protein measurements, 20 L4s were picked from OP50 maintenance plates and allowed to mature for 24h on HTT15 bacteria carrying empty pL4440 vector, picked into 100 μl of 0.05% Tween-20, the bacteria removed by centrifugation and the worms lysed with glass beads in a Ribolyser. Protein concentration was determined against a BSA standard as for the fly. 10-20 repeats were performed for each sample and 5% lowest and 5% highest measurements removed from each group.

Statistical Analysis

Mixed Effect Linear Models, Linear Models, Generalised Linear Models, post-hoc tests and Log-rank comparisons were performed in JMP (SAS), all other analysis in R. MECPH analysis was performed using the *coxme* package (Therneau, T., 2012, http://CRAN.R-project.org/package=coxme), CPH using *survival* (Therneau, T., 2013, http://CRAN.R-project.org/package=survival), Mixed Effects Ordinal Logistic analysis using

ordinal (Christensen, R. H. B., 2012 http://www.cran.r-project.org/package=ordinal). Further details are given in **Table 1**. To determine difference in slopes of the regression lines between the two gene sets in Figure 2A, Linear Model was fitted with RU486-induced response in *dfoxo*Δ/Δ S_1*106>dfoxo* as the dependant variable and the response in S_1*106>dfoxo* (continuous) and gene set (categorical) as explanatory variables, testing for the significance of the interaction term.

Supplemental References

Benjamini, Y., and Hochberg, Y. (1995). Controlling the False Discovery Rate: A practical and Powerful Approach to Multiple Testing. J. R. Stat. Soc. Ser. B Stat. Methodol. 57, 289-300.

Bolstad, B.M., Irizarry, R.A., Astrand, M., and Speed, T.P. (2003). A comparison of normalization methods for high density oligonucleotide array data based on variance and bias. Bioinformatics 19, 185-193.

Dennis, G., Jr., Sherman, B.T., Hosack, D.A., Yang, J., Gao, W., Lane, H.C., and Lempicki, R.A. (2003). DAVID: Database for Annotation, Visualization, and Integrated Discovery. Genome Biol 4, P3.

Gronke, S., Clarke, D.-F., Broughton, S., Andrews, T.D., and Partridge, L. (2010). Molecular evolution and functional characterisation of Drosophila insulin-like peptides. PLoS Genet 6, e1000857.

Ikeya, T., Galic, M., Belawat, P., Nairz, K., and Hafen, E. (2002). Nutrient-dependent expression of insulin-like peptides from neuroendocrine cells in the CNS contributes to growth regulation in Drosophila. Curr Biol 12, 1293-1300.

Irizarry, R.A., Bolstad, B.M., Collin, F., Cope, L.M., Hobbs, B., and Speed, T.P. (2003a). Summaries of Affymetrix GeneChip probe level data. Nucleic Acids Res 31, e15.

Irizarry, R.A., Hobbs, B., Collin, F., Beazer-Barclay, Y.D., Antonellis, K.J., Scherf, U., and Speed, T.P. (2003b). Exploration, normalization, and summaries of high density oligonucleotide array probe level data. Biostatistics 4, 249-264.

Lin, K., Hsin, H., Libina, N., and Kenyon, C. (2001). Regulation of the Caenorhabditis elegans longevity protein DAF-16 by insulin/IGF-1 and germline signaling. Nat Genet 28, 139-145.

Love, D.C., Ghosh, S., Mondoux, M.A., Fukushige, T., Wang, P., Wilson, M.A., Iser, W.B., Wolkow, C.A., Krause, M.W., and Hanover, J.A. (2010). Dynamic O-GlcNAc cycling at promoters of Caenorhabditis elegans genes regulating longevity, stress, and immunity. Proc Natl Acad Sci U S A 107, 7413-7418.
