# Supplementary material for: Cell-Nonautonomous Effects of dFOXO/DAF-16 in Aging
Source: Cell Rep. 2014 Feb 6;6(4):608–16. doi: 10.1016/j.celrep.2014.01.015 (PMC3969275; doi:10.1016/j.celrep.2014.01.015)
Supplement: Document S2. Article plus Supplemental Information [file mmc3.pdf]

Cell PRESS
Open ACCESS

Cell Reports
Report

Cell-Nonautonomous Effects of dFOXO/DAF-16 in Aging

Nazif Alic,[1] Jennifer M. Tullet,[1] Teresa Niccoli,[1] Susan Broughton,[1,3] Matthew P. Hoddinott,[1,2] Cathy Slack,[1] David Gems,[1] and Linda Partridge[1,2,*]
[1]Department of Genetics, Evolution and Environment, Institute of Healthy Ageing, University College London, Darwin Building, Gower Street, London WC1E 6BT, UK
[2]Max Planck Institute for Biology of Ageing, Joseph-Stelzmann-Strasse 9b, 50931 Cologne, Germany
[3]Present address: Division of Biomedical and Life Sciences, Faculty of Health and Medicine, Lancaster University, Lancaster LA1 4YQ, UK
*Correspondence: l.partridge@ucl.ac.uk
http://dx.doi.org/10.1016/j.celrep.2014.01.015
This is an open-access article distributed under the terms of the Creative Commons Attribution License, which permits unrestricted use, distribution, and reproduction in any medium, provided the original author and source are credited.

SUMMARY

***Drosophila melanogaster* and *Caenorhabditis elegans* each carry a single representative of the Forkhead box O (FoxO) family of transcription factors, dFOXO and DAF-16, respectively. Both are required for lifespan extension by reduced insulin/Igf signaling, and their activation in key tissues can extend lifespan. Aging of these tissues may limit lifespan. Alternatively, FoxOs may promote longevity cell nonautonomously by signaling to themselves (FoxO to FoxO) or other factors (FoxO to other) in distal tissues. Here, we show that activation of dFOXO and DAF-16 in the gut/fat body does not require *dfoxo/daf-16* elsewhere to extend lifespan. Rather, in *Drosophila*, activation of dFOXO in the gut/fat body or in neuroendocrine cells acts on other organs to promote healthy aging by signaling to other, as-yet-unidentified factors. Whereas FoxO-to-FoxO signaling appears to be required for metabolic homeostasis, our results pinpoint FoxO-to-other signaling as an important mechanism through which localized FoxO activity ameliorates aging.**

INTRODUCTION

Forkhead box O (FoxO) transcription factors (TFs) are involved in a plethora of cellular processes to regulate whole-organism physiology and are major determinants of animal lifespan (Partridge and Brüning, 2008; Salih and Brunet, 2008). Activation of FoxO-family TFs mediates the lifespan-extending effects of dampened insulin/insulin-like growth factor-like signaling (IIS) in both worms and flies (Kenyon et al., 1993; Slack et al., 2011; Yamamoto and Tatar, 2011). This evolutionary conservation appears to extend to humans, because certain genetic variants of *Foxo3A* are robustly associated with human longevity (Flachsbart et al., 2009; Kuningas et al., 2007; Willcox et al., 2008). Indeed, Forkhead-like TFs can even extend lifespan in a single-celled eukaryote, budding yeast (Postnikoff et al., 2012).

In *Drosophila melanogaster*, tissue-restricted activation of *Drosophila foxo* (*dfoxo*) is sufficient to extend lifespan (Demontis and Perrimon, 2010; Giannakou et al., 2004; Hwangbo et al., 2004). Such an increase in *dfoxo* activity confined to key tissues could promote whole-organism survival in two mutually compatible ways: cell autonomously and cell nonautonomously. The lifespan of the animal could be limited by pathology in a particular organ, so that cell-autonomous action of *dfoxo* in that organ alone could promote longevity (Rera et al., 2013). In addition, healthy aging may involve the coordinated action of multiple organ systems, with *dfoxo* in one organ altering whole-organism physiology through systemic changes (Demontis and Perrimon, 2010; Hwangbo et al., 2004; Rera et al., 2013). For example, adult-onset induction of *dfoxo* in the midgut and abdominal fat body (equivalent to mammalian liver and adipose) activates the transcription of *Drosophila insulin-like peptide* (*dilp*) *6* in the fat body, whereas in muscle *dfoxo* represses the activin ligand *dowdle*, and these endocrine signals have a distal effect on the median neurosecretory cells (mNSCs) in the brain, resulting in lowered DILP2 peptide in circulation (Bai et al., 2012, 2013). Importantly, upregulation of *dilp6* is required for the beneficial effect of *dfoxo* on lifespan (Bai et al., 2012). However, whether this requires *dfoxo* in tissues other than the ones producing the DILP6 signal remains unexamined.

The single *Caenorhabditis elegans* FoxO ortholog, DAF-16, can act both cell autonomously and cell nonautonomously to regulate gene expression (Libina et al., 2003; Murphy et al., 2007; Qi et al., 2012; Zhang et al., 2013). DAF-16 activity in one tissue can induce DAF-16 activity in another in a process of tissue entrainment mediated by altered expression of an insulin-like peptide (Murphy et al., 2007), which is highly reminiscent of the situation in the fly. For this reason, it has been widely believed that the fruit fly's *dfoxo* acts from specific cells to activate dFOXO in the whole animal in an instance of *dfoxo*-to-*dfoxo* signaling (Bai et al., 2012, 2013; Demontis and Perrimon, 2010; Hwangbo et al., 2004). However, the relevance of this tissue entrainment for *Drosophila* lifespan has not been experimentally tested. Indeed, there is a growing awareness that FoxOs in one tissue can also signal to other factors elsewhere, i.e., FoxO-to-other signaling. In the worm, DAF-16 activity in one tissue can elicit *daf-16*-independent responses in the receiving tissues (Qi et al., 2012; Zhang et al., 2013). The existence and relevance of *dfoxo*-to-other intertissue signaling is unexplored in *Drosophila*.

Here, we establish the relevance to aging of the cell-nonautonomous effects of *dfoxo*, differentiating between *dfoxo*-to-*dfoxo*

and *dfoxo*-to-other signaling in adult *Drosophila*. We find that *dfoxo*-to-*dfoxo* signaling does not affect aging and confirm that the same is true of the worm *daf-16*. On the other hand, *dfoxo* in the gut and fat body can promote health of the neuromuscular system, possibly via transcriptional regulation of a secreted neuropeptide-like molecule, and *dfoxo* in mNSCs can extend lifespan. Both effects are independent of *dfoxo*'s presence in other tissues, demonstrating the relevance of *dfoxo*-to-other signaling in *Drosophila* aging. At the same time, *dfoxo*-to-*dfoxo* signaling is required for the metabolic effects of localized *dfoxo* induction, showing that distinct physiological effects of tissue-restricted *dfoxo* activation are mediated by different signaling routes.

RESULTS

dfoxo-to-*dfoxo* Signaling in *Drosophila* Is Dispensable for Extension of Lifespan by Gut/Fat Body or mNSC *dfoxo*

To examine whether activation of dFOXO in other tissues contributes to the lifespan-extending effects of induction of *dfoxo* in the adult gut and fat body, we generated strains where the tissue-restricted induction of *dfoxo* could be triggered by the RU486 inducer in either an otherwise wild-type or a *dfoxo*-null background (S_1106>*dfoxo* or *dfoxo*Δ/Δ S_1106>*dfoxo*). We used females, where the effects of *dfoxo* activation on aging are clearly observed (Giannakou et al., 2004). Because the lifespan effects of ectopic *dfoxo* expression can be conditional on the nutritional status of the animal (Bai et al., 2012; Min et al., 2008), we used a food with the optimal amount of dietary yeast (10% weight/volume) for lifespan under our laboratory conditions (Bass et al., 2007) and where expression of *dfoxo* targeted to adult gut and fat body robustly extends lifespan (Giannakou et al., 2008). Importantly, on this food, lifespan is maximized so that the effects of *dfoxo* can be studied as additional to the beneficial effects of the diet.

We found no detectable expression of dFOXO protein or of *dfoxo* transcript in the *dfoxo*Δ/Δ S_1106>*dfoxo* females in the absence of the inducer (Figures 1A and 1B). Feeding RU486 for 5 days resulted in equivalent increases in *dfoxo* transcript in S_1106>*dfoxo* and *dfoxo*Δ/Δ S_1106>*dfoxo* females (Figure 1B; see Table 1 for detailed statistical analysis). The S_1106 driver has been thoroughly characterized and, in the female fly, only drives expression in the gut and fat body (Poirier et al., 2008). To ensure the flies are experiencing the same nutritional conditions, we examined their feeding behavior with the proboscis-extension assay (Wong et al., 2009) and found no significant differences (Figure S1A).

We examined the effect on lifespan resulting from the presence of the inducer in the S_1106>*dfoxo* and *dfoxo*Δ/Δ S_1106>*dfoxo* lines in two sequential, independent, experimental trials (Figure 1C), recording deaths of over 1,000 flies in total. The presence of RU486 from day 2 of adulthood extended the median lifespan of S_1106>*dfoxo* females on average by 6% (log-rank test $p < 0.05$ for each trial; Figure 1C). The magnitude of the effect was less than previously reported (Giannakou et al., 2004) but is consistent with more recent work in our laboratory (Giannakou et al., 2008) and with six other independent trials performed in the course of the last 4 years (2008–2012, average median extension 5%; Figure S1B). The lifespan of *dfoxo*-null flies was also extended by a similar percentage (average 10%, log-rank test $p < 0.05$ for each trial; Figure 1C). Thus, the presence of *dfoxo* in the rest of the body is not required for the lifespan-extending effects of its induction in the gut/fat body.

Flies lacking *dfoxo* have short lifespans (Figure 1C), possibly due to developmental effects of the mutation (Slack et al., 2011), complicating the direct comparison between effects of RU486 in the two lines. Cox proportional hazards (CPH) is a survival analysis that allows for the significance of several covariates and their interactions to be examined. To establish whether there was any statistically significant difference in the response of *dfoxo*-null and wild-type flies to RU486, we combined the two experimental trials and analyzed the survival data using a mixed-effects Cox proportional hazards (MECPH) model (Table 1). Both RU486 (30% reduction in risk of death, $p = 2 \times 10^{-4}$) and the presence of genomic *dfoxo* ($p < 10^{-15}$) had a significant effect on lifespan, but their interaction did not ($p = 0.95$). The absence of a significant interaction confirms that the effect of RU486 did not differ between the lines and hence that the presence of *dfoxo* elsewhere in the body does not affect the extension of lifespan by induction of *dfoxo* in the gut and fat body. Thus, tissue entrainment through *dfoxo*-to-*dfoxo* signaling is not required for longevity.

This result indicated that either *dfoxo* acts cell autonomously to extend lifespan or that *dfoxo* in one tissue activates *dfoxo*-independent longevity-assurance mechanisms in other tissues. The latter would occur through dFOXO-to-other signaling, as has been observed for DAF-16 (Qi et al., 2012; Zhang et al., 2013). To further test for *dfoxo*-to-other signaling, we manipulated the levels of *dfoxo* in cells whose prominent function is in adult endocrine signaling. mNSCs in the adult brain play an important role in aging by producing DILP2, DILP3, and DILP5 (Broughton et al., 2005) and possibly other endocrine signals. Expressing *dfoxo* specifically in the mNSC, using a *dilp2-GAL4* driver, extended the lifespan of female flies in both wild-type and *dfoxo* nulls ($p < 0.01$ to either control in both backgrounds; Figure 1D; Table 1). CPH analysis found significant effects of the genomic *dfoxo* ($p < 10^{-15}$) and its induction in *dilp2GAL4*>*dfoxo* flies (50% reduction in risk of death, $p = 2.2 \times 10^{-5}$) on lifespan but no evidence for a significant interaction between them ($p = 0.33$; Table 1). This confirmed that the effect on lifespan is independent of *dfoxo* in tissues other than the mNSCs.

This longevity phenotype must represent a gain of function in the mNSC, because the ablation of mNSCs, representing a loss of function in these cells, requires *dfoxo* to extend lifespan (Slack et al., 2011). Indeed, we observed no significant changes in the mRNA levels of *dilp2*, *dilp3*, and *dilp5* upon induction of *dfoxo* in mNSCs (Figures S1C and S1D). Furthermore, we found no changes in the mRNA levels of any *dilp*s detectable in whole adults (*dilp2* through to *dilp7*), including *dilp6* (Figure S1D), or their binding partner and regulator, *Imp-L2* (Alic et al., 2011b) (Figure S1E), upon activation of *dfoxo* in the mNSCs, confirming that *dilp2GAL4* > *dfoxo* flies are not experiencing any alterations in systemic IIS activity. Because the principal role of these cells is

Cell
PRESS
Open
ACCESS

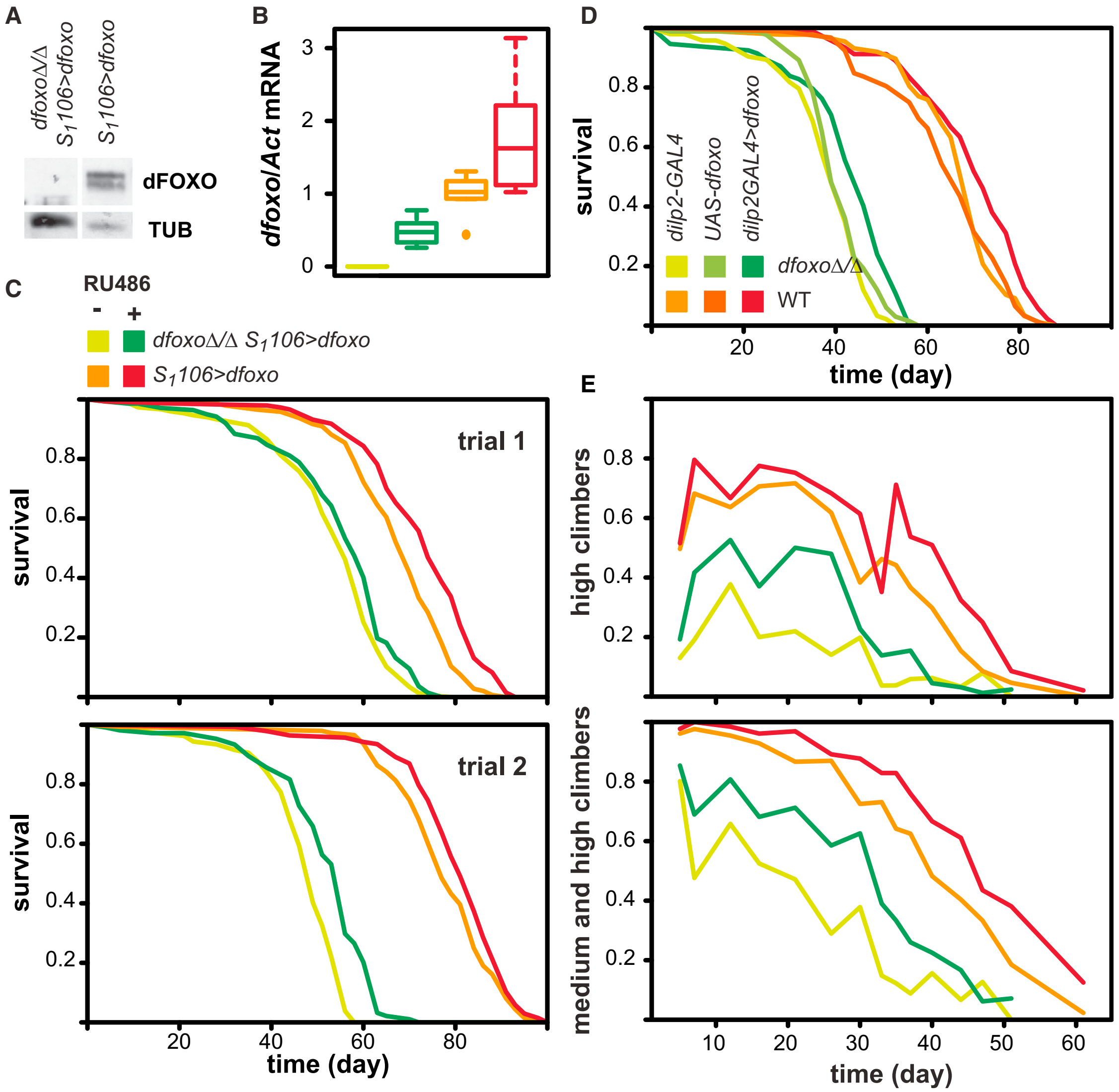

Figure 1. ***dfoxo*-to-*dfoxo* Signaling Is Not Required for the Antiaging Effects of Increased dFOXO Activity in the Gut/Fat Body or mNSC**

(A) Western blots of dFOXO and the tubulin loading control on whole-fly protein extracts from *$S_1$106>dfoxo* or *dfoxoΔ/Δ $S_1$106>dfoxo* female flies in the absence of the inducer.

(B) *dfoxo* transcript levels (relative to *Act* and with *$S_1$106>dfoxo* -RU486 set to 1) in *$S_1$106>dfoxo* or *dfoxoΔ/Δ $S_1$106>dfoxo* female flies fed or not RU486.

(C) Survival of *$S_1$106>dfoxo* or *dfoxoΔ/Δ $S_1$106>dfoxo* female flies in presence or absence of RU486 determined in two experimental trials (top and bottom panel).

(D) Survival of *dilp2GAL4>dfoxo* female flies, or the two genetic controls (*dilp2-GAL4* or *UAS-dfoxo* alone), in wild-type (WT) or *dfoxoΔ/Δ* backgrounds.

(E) The proportion of high climbers (top panel) or combined medium and high climbers (bottom panel) in three cohorts (combined) of *$S_1$106>dfoxo* or *dfoxoΔ/Δ $S_1$106>dfoxo* female flies in the presence or absence of RU486.

Note the same color code is used in (B), (C), and (E) and is given in (C). See Table 1 for statistical analysis of data in (B)–(E). Where used, box plots indicate median, first and third quartile, data range, and outliers.See also Figure S1.

in endocrine signaling, the physiological effects of *dfoxo* activation in the mNSCs are most likely to be mediated by *dfoxo*-to-other signaling.

Gut/Fat Body *dfoxo* Acts at a Distance Independently of *dfoxo* in Target Tissues

To further investigate the role of *dfoxo*-to-other signaling in fly physiology, we examined the beneficial effects of gut/fat body induction of *dfoxo* on the neuromuscular system, an organ system distal to the site of *dfoxo* activation in our model. The ability of flies to climb a vertical surface is a suitable physiological measure of the performance of this organ system and is susceptible to aging (Cook-Wiens and Grotewiel, 2002). We scored the number of low, medium, and high climbers in three cohorts of ~15 individuals of *$S_1$106>dfoxo* or *dfoxoΔ/Δ $S_1$106>dfoxo* genotype in the presence or absence of RU486 over ~10 weeks.

Induction of *dfoxo* expression in the gut and fat body enhanced the climbing ability of female flies throughout their lifespan, observed as an increase in the proportion of high, or combined medium and high, climbers (Figure 1E). This enhancement could be seen in both the wild-type and *dfoxo*-null backgrounds, revealing that it is independent of *dfoxo* in other tissues. Indeed, statistical analysis (mixed-effects ordinal logistic model, Table 1) confirmed that the effect of RU486 ($p = 1.8 \times 10^{-3}$) and *dfoxo* ($p = 5.4 \times 10^{-15}$) were both significant but that their interaction was not ($p = 0.12$). Hence, local action of *dfoxo* in the gut and fat body has a beneficial effect on the performance of a distal organ system. This could occur through systemic effects of healthy gut and fat body or through specific signaling events. In the latter case, its independence from *dfoxo* in the distal cells is again consistent with *dfoxo*-to-other signaling.

Gut/Fat Body *dfoxo* Regulates Expression of *Nplp4*

In order to trigger *dfoxo*-to-other signaling, the gut/fat body *dfoxo* may regulate the expression of a secreted factor other than *dilp6*. To identify such a factor, we determined the whole-fly, genome-wide, transcriptional changes induced by RU486 in the S_1106>*dfoxo* flies (Table S1; Figure 2A). We found that, besides the documented changes in *dilp6* (Bai et al., 2012), induction of the gut/fat body *dfoxo* altered the mRNA levels of another gene encoding a signal peptide targeting its protein product for secretion, *neuropeptide-like precursor 4* (*Nplp4*). The mature product of this gene is a YSY peptide of previously unknown function (Nässel and Winther, 2010). Quantitative PCR confirmed that activation of *dfoxo* led to repression of this gene (Figure 2B). Hence, *Nplp4* is a candidate for a secreted factor mediating *dfoxo*-to-other signaling. Interestingly, this gene was repressed in both heads and bodies of S_1106>*dfoxo* females fed RU486 ($p = 0.048$ for RU486, $p = 0.19$ for body part:RU486 interaction; Figure 2B; Table 1), whereas, as expected, the induction of *dfoxo* itself was confined to the body, ($p = 0.053$ for body part:RU486 interaction; Figure 2C; Table 1), indicating *Nplp4* responds to dFOXO both locally and distally.

Importance of *dfoxo*-to-*dfoxo* Signaling to *Drosophila* Metabolism

Although *dfoxo*-to-*dfoxo* signaling is not required for lifespan extension, it may be required for other physiological changes in response to the activation of dFOXO in gut and fat body. To query the existence of these other physiological effects, we examined whether there are transcriptional changes in response to RU486 in S_1106>*dfoxo* flies that do not occur in *dfoxo*Δ/Δ S_1106>*dfoxo* females. We reasoned that the genes and processes that respond to RU486 in S_1106>*dfoxo* flies but fail to do so in the *dfoxo*Δ/Δ S_1106>*dfoxo* females may be regulated through *dfoxo*-to-*dfoxo* signaling.

Among the genes regulated by RU486 in S_1106>*dfoxo* females, we identified all those for which the RU486-induced transcriptional change was altered by mutation of *dfoxo* by finding the genes whose transcript levels show a significant interaction between the presence of genomic *dfoxo* and its induction by RU486 in the relevant linear model (Figure 2A; Table S1). The magnitude of fold-change for these genes was reduced on average in *dfoxo*Δ/Δ S_1106>*dfoxo* compared to S_1106>*dfoxo* females (Figure 2A), indicating they require the presence of genomic *dfoxo* for correct expression. We confirmed the significance of this effect using a linear model ($p = 1 \times 10^{-7}$; Figure 2A and the associated caption). Note that *Nplp4* was equally repressed in *dfoxo*Δ/Δ S_1106>*dfoxo* and S_1106>*dfoxo* females (Table S1).

Examination of the Gene Ontology categories enriched in this group of genes revealed "proteolysis" as overrepresented ($p = 3.1 \times 10^{-7}$; Table S1), hinting that protein metabolism may be regulated through a *dfoxo*-to-*dfoxo* signal. Pursuing this lead, we found that RU486 feeding triggered a small (12%) but significant reduction in total protein content of S_1106>*dfoxo* females and that this effect was blocked by deletion of *dfoxo* (Figure 2D; $p = 3.1 \times 10^{-3}$ for RU486 by genotype interaction; Table 1). Similar significant changes were not observed in total triglyceride, total trehalose, or total glycogen content (Figure S2A). However, due to assay variability, we cannot discount possible subtle changes in these metabolites. On the other hand, total body mass followed closely the protein content (Figure 2E; Table 1).

Surprisingly, both deletion of *dfoxo* and its induction in the gut and fat body reduced total protein content and fly weight. The two manipulations may act in different ways. The small size of *dfoxo* nulls is due to the developmental effects of the mutation (Slack et al., 2011) and, together with their reduced fecundity (Slack et al., 2011) (Figure S2B), could explain the lowered body weight and protein content. On the other hand, S_1106>*dfoxo* was induced in adulthood and had no effect on fecundity in either wild-type or *dfoxo*-null females (Figure S2B), but it had an effect on body weight and protein content. Hence, the latter two metabolic phenotypes of dFOXO activation in gut/fat body may depend on *dfoxo*-to-*dfoxo* signaling. However, because *dfoxo* was absent in all tissues throughout development, we cannot exclude the possibility that the inability of *dfoxo* nulls to respond to RU486, for these traits, is due to developmentally altered gut/fat body function. Note that the expression pattern of the proteolysis genes, which initially led us to this phenotype, cannot mechanistically explain the loss of protein in S_1106>*dfoxo* females upon RU486 feeding, because these genes are repressed in this condition (Figure S2C), but may rather be part of a homeostatic mechanism. Nevertheless, the results strongly indicate the effects on lifespan and metabolism of tissue-restricted activation of dFOXO can be separated by their requirement for *dfoxo*-to-other or *dfoxo*-to-*dfoxo* signaling.

daf-16-to-*daf-16* Signaling in *C. elegans* Is Dispensable for Extension of Lifespan by Gut *daf-16*

The worm intestine serves a functionally similar role to the gut and fat body in *Drosophila*. It is an important *daf-16* signaling center, and increased DAF-16 activity in this organ activates DAF-16 elsewhere (Libina et al., 2003; Murphy et al., 2007). However, in the context of reduced IIS resulting from mutation in *daf-2*, *daf-16* presence solely in the intestine is sufficient substantially, but not completely, to restore the *daf-2* mutant longevity in otherwise *daf-16*-deficient worms (Libina et al., 2003). This indicates that the observable *daf-16*-to-*daf-16* signaling is not essential for lifespan extension. However, the

Table 1. Statistical Analysis

Relevant Figure	Model and Description	Random Effect	Coefficient[a]	Estimate[b]	SE	p Value
Figure 1B	mixed-effects linear					
	n = 7–8	batch	intercept	0.81	0.15	0.11
			dfoxo	0.571	7.1×10^{-2}	$<10^{-4}$
			RU486	0.311	7.1×10^{-2}	2.0×10^{-4}
			dfoxo:RU486	6.6×10^{-2}	7.1×10^{-2}	0.36
Figure 1C	MECPH					
	1,050 deaths (1,078 total)	experimental trial	*dfoxo*	−2.1	0.11	$<10^{-15}$
			RU486	−0.34	9.1×10^{-2}	2×10^{-4}
			dfoxo:RU486	8.5×10^{-3}	0.13	0.95
Figure 1D	CPH					
	533 deaths (545 total)	NA	*dfoxo*	−3.26	0.22	$<10^{-15}$
			UAS-dfoxo	−0.23	0.15	0.13
			dilp2GAL4>dfoxo	−0.65	0.15	2.2×10^{-5}
			dfoxo:UAS-dfoxo	0.28	0.21	0.19
			dfoxo:dilp2GAL4>dfoxo	0.21	0.22	0.33
Figure 1E	mixed-effects ordinal logistic					
	total observations = 2,179	biological repeat (vial)	time	-7.1×10^{-2}	7.8×10^{-3}	$<10^{-15}$
			dfoxo	2.1	0.27	5.4×10^{-15}
			RU486	0.81	0.26	1.8×10^{-3}
			time:*dfoxo*	-5.4×10^{-3}	9.8×10^{-3}	0.58
			time:RU486	2.9×10^{-3}	0.01	0.78
			dfoxo:RU486	−0.59	0.38	0.12
			time:*dfoxo*:RU486	8.1×10^{-3}	1.3×10^{-2}	0.54
Figure 2B	linear					
	n = 5	NA	intercept	3.4	0.25	$<10^{-4}$
			head	2.6	0.25	$<10^{-4}$
			RU486	−0.54	0.25	4.8×10^{-2}
			head:RU486	−0.34	0.25	0.19
Figure 2C	linear					
	n = 5	NA	intercept	2.8	0.25	$<10^{-4}$
			head	1.2	0.25	$<10^{-4}$
			RU486	0.38	0.25	1.8×10^{-2}
			head:RU486	−0.28	0.25	5.4×10^{-2}
Figure 2D	mixed-effects linear					
	n = 8–10	batch	intercept	0.12	1.4×10^{-2}	6.8×10^{-2}
			dfoxo	1.7×10^{-2}	1.8×10^{-3}	$<10^{-4}$
			RU486	-5.5×10^{-3}	1.8×10^{-3}	4×10^{-3}
			dfoxo:RU486	-5.7×10^{-3}	1.8×10^{-3}	3.1×10^{-3}
Figure 2E	mixed-effects linear					
	n = 27–30	batch	intercept	1.6	0.03	$<10^{-4}$
			dfoxo	9.6×10^{-2}	1.3×10^{-2}	$<10^{-4}$
			RU486	-3.8×10^{-2}	1.3×10^{-2}	3.3×10^{-3}
			dfoxo:RU486	-2.6×10^{-2}	1.3×10^{-2}	0.04
Figures 3A and S3A	MECPH					
	944 deaths (1,128 total)	experimental trial	*daf-16*	−2.5	0.11	$<10^{-15}$
			intestinal *daf-16*	−1.3	0.10	$<10^{-15}$
			daf-16:intestinal *daf-16*	1.1	0.14	2×10^{-15}

(*Continued on next page*)

Table 1. Continued

Relevant Figure	Model and Description	Random Effect	Coefficient[a]	Estimate[b]	SE	p Value
Figure 3B	linear					
	n = 8–16	NA	intercept	0.52	7.9×10^{-3}	$<10^{-4}$
			daf-16	-2.3×10^{-2}	7.9×10^{-3}	$<10^{-4}$
			intestinal *daf-16*	-3.7×10^{-2}	7.9×10^{-3}	6.2×10^{-3}
			daf-16:intestinal *daf-16*	-9.3×10^{-3}	7.9×10^{-3}	0.24

[a]In all models, the effect of presence of *dfoxo* (*daf-16*) or its induction (overexpression) is examined; *dilp2-GAL4* was used as reference for *dilp2GAL4>dfoxo* and *UAS-dfoxo*; ‘‘body’’ was used as reference for body versus head comparisons; ‘‘:’’ indicates interaction term.

[b]For mixed-effect linear models and linear models, the coefficient estimates either have no units because they are derived from the *dfoxo/Act* or *Nplp4/Act* transcript ratios (for Figures 1B, 2B, and 2C) or are given in mg (for Figures 2D and 2E) or μg (Figure 3B). For MECPH and CPH models, the coefficient estimate is the natural log of the hazard ratio, where a negative value indicates a beneficial effect on survival. For mixed-effect ordinal logistic, these are log-transformed odds of climbing high, where a negative value indicates a reduction in climbing ability.

question remains whether *daf-16*-to-*daf-16* signaling contributes to the longevity promoted by DAF-16 alone.

To investigate this, we tested for a role of *daf-16*-to-*daf-16* signaling in longevity induced by *daf-16* overexpression in the intestine. Increasing DAF-16 activity alone, within otherwise wild-type worms, has two opposing effects on lifespan. DAF-16 stimulates germline hyperplasia in a cell-nonautonomous manner (Qi et al., 2012). This shortens the animal’s lifespan and masks the second, prolongevity effect of DAF-16 (Qi et al., 2012). Indeed, semiubiquitous overexpression of *daf-16* extends lifespan only if the germline cell proliferation is blocked, e.g., by administration of 5-fluoro-2′-deoxyuridine (FUdR) (Qi et al., 2012).

Similar to Qi and coworkers, we used FUdR to reveal the lifespan extension caused by overexpression of *daf-16* from the intestinal-specific *ges-1* promoter in otherwise wild-type worms and asked whether this effect required the presence of *daf-16* in other tissues (Figure 3A). We found that intestinal activation of DAF-16 can extend lifespan of both wild-type and *daf-16*-deficient (*daf-16(mu86)*) worms fed HT115 bacteria (log-rank $p < 0.05$ in three out of five and three out of three assays, respectively; Figures 3A and S3A). We confirmed these findings using a second, independently derived transgene (Figure S3B).

Upon further examination, we found that the ability of the intestinal *daf-16* to extend wild-type lifespan was conditional on the food source and had a small but opposite effect when worms were fed on OP50 bacteria (Figure S3C). This is similar to the effect of gut/fat body expression of *dfoxo* on *Drosophila* lifespan, which can depend on available nutrition (Bai et al., 2012; Min et al., 2008). Importantly, however, the ability of the intestinal *daf-16* to extend lifespan in the absence of *daf-16* elsewhere was observed under all conditions, including the worms fed OP50 (Figures 3 and S3A–S3C). MECPH analysis of the combined data obtained with one of the transgenes on HT115 bacteria (Figures 3 and S3A) confirmed that both the effects of *daf-16* presence in the whole worm and its intestinal induction were significant ($p < 10^{-15}$ for both) and revealed a significant interaction of the two main effects ($p = 2 \times 10^{-15}$; Table 1). Thus, intestinal *daf-16* extended the lifespan of the mutant more than that of the wild-type worms (Figure 3), confirming that, as in the fly (Figure 1C) and during IIS dampening in the worm (Libina et al., 2003), tissue entrainment through *daf-16*-to-*daf-16* signaling is not required for lifespan extension and, indeed, could even have the opposite effect.

Prompted by the findings in the fly (Figure 2D), we also examined if the induction of intestinal *daf-16* in worms had an effect on their total protein content. We found that the intestinal *daf-16* reduced whole-worm protein content ($p < 10^{-4}$; Figure 3B; Table 1) on HTT15 bacteria. In contrast to the fly, we found no evidence that this reduction is prevented by the absence of *daf-16* in other tissues, and, in fact, found that mutation of *daf-16* increases the overall protein content (Figure 3B; Table 1). We obtained similar results with the second transgene (Figure S3D). Hence, this phenotype is mediated either cell autonomously by *daf-16* in the intestine or through *daf-16*-to-other signaling. Thus, whereas the physiological effect appears conserved between the fly and worm, the way it is mediated differs.

It is also of note that, similar to the lifespan effect of intestinal *daf-16* in an otherwise wild-type worm, we found this modulation of protein content conditional on the bacterial food source and neither the transgenes nor mutation of *daf-16* had any significant effect when worms were fed OP50 bacteria (data not shown). For both lifespan and protein content, the alteration of phenotype between OP50 and HTT15 is reminiscent of the lifespan effects of certain sensory mutants in *C. elegans* (Maier et al., 2010) and suggests that intestinal DAF-16 plays a role in food perception.

DISCUSSION

In the fly, tissue-restricted dFOXO triggers endocrine factors to cause a drop in overall, systemic, IIS activity (Bai et al., 2012, 2013; Demontis and Perrimon, 2010; Hwangbo et al., 2004). Because insulin signals repress the activity of FoxOs (Brunet et al., 1999), this will result in body-wide activation of dFOXO (tissue entrainment), including further activation of dFOXO in the specific tissue (positive feedback). Our results show that the tissue entrainment is not required for the beneficial effects of *dfoxo* on lifespan or on healthspan. The regulation of systemic IIS by local *dfoxo* can still be relevant to lifespan as part of a positive feedback loop. For example, the upregulation of *dilp6* by dFOXO in the fat body triggers a reduction in global IIS activity, and this, in turn, could be affecting lifespan by fine-tuning the activity of dFOXO in the fat body itself.

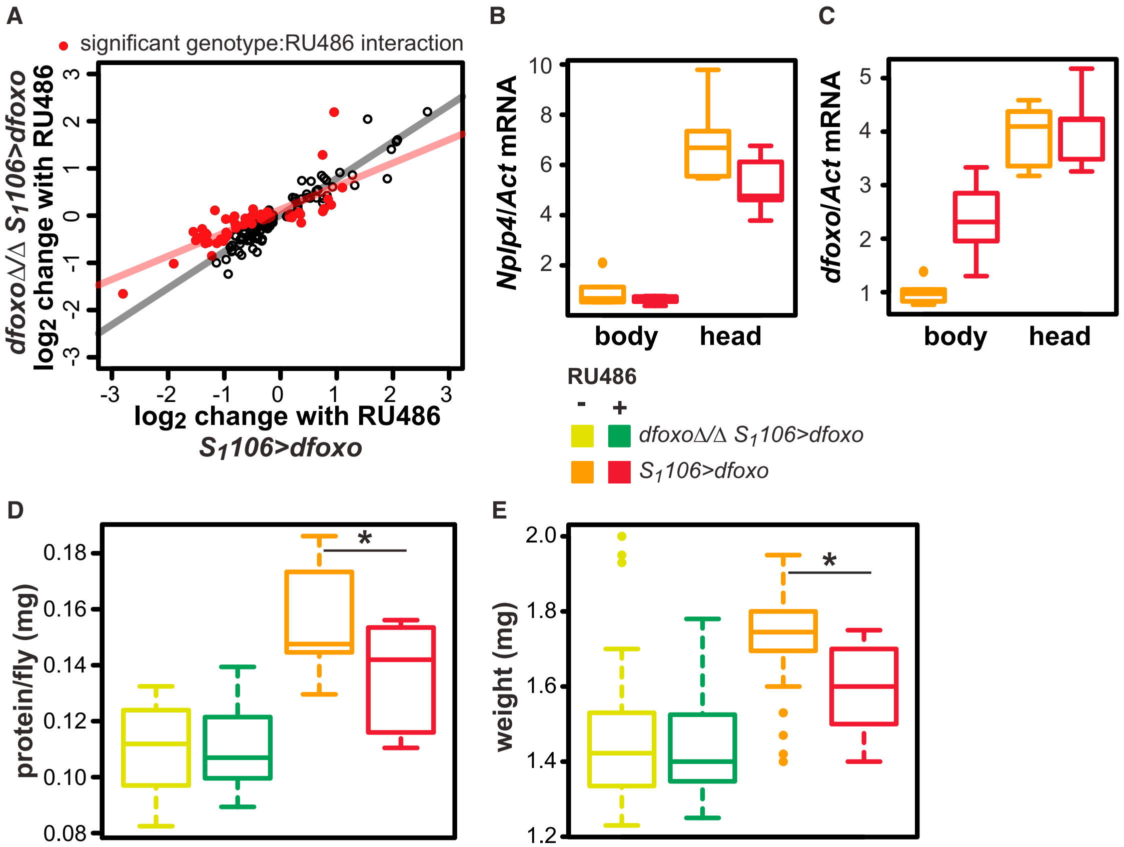

Figure 2. Transcriptional Regulation by Gut/Fat Body *dfoxo* and the Physiological Relevance of *dfoxo*-to-*dfoxo* Signaling

(A) Log_2 fold change in transcript levels upon RU486 administration in S_1106>*dfoxo* (x axis) or *dfoxoΔ/Δ* S_1106>*dfoxo* (y axis) female flies for the genes significantly changed in S_1106>*dfoxo*. Red indicates the genes whose response to RU486 is significantly altered by genotype (significant genotype:RU486 interaction in the linear model). The red line is the regression line for these genes (slope = 0.49) and the black line is for the others (slope = 0.77). The significant difference in slope ($p = 1 \times 10^{-7}$) indicates the genes marked in red are overall less responsive to RU486 in *dfoxoΔ/Δ* S_1106>*dfoxo* than in S_1106>*dfoxo* female flies. Gene lists are given in Table S1.

(B) *Nplp4* transcript levels (relative to *Act* and with body -RU486 set to 1) in bodies or heads of S_1106>*dfoxo* female flies fed or not RU486.

(C) *dfoxo* transcript levels (relative to *Act* and with body -RU486 set to 1) in bodies or heads of S_1106>*dfoxo* female flies fed or not RU486.

(D) Protein content of individual S_1106>*dfoxo* or *dfoxoΔ/Δ* S_1106>*dfoxo* female flies after 5-day feeding with RU486 or not.

(E) Individual fly weight for the same conditions.

Note the same color code is used in (B)–(E) and is given in (B). In (D) and (E), asterisk indicates significant difference at $p < 0.05$ by post hoc, pair-wise t test between − and + RU486 conditions. See Table 1 for statistical analysis of data in (B)–(E). See Figure S2 for further data.

Under certain experimental conditions, the lifespan effects of ectopic *dfoxo* expression can be conditional on the nutrients available to the animal (Bai et al., 2012; Min et al., 2008). Hence, tissue entrainment may also have conditional relevance. In addition, our results indicate that *dfoxo*-to-*dfoxo* signaling is required for the metabolic effects of localized *dfoxo* induction, namely a drop in protein content and fly weight, and further examination may reveal roles for *dfoxo*-to-*dfoxo* signals in yet other aspects of physiology.

Both DAF-16 in *C. elegans* and dFOXO in *Drosophila* can extend lifespan from the gut/fat body without being present in other tissues. The gut and/or fat body may represent the organs most vulnerable to aging, so that DAF-16/dFOXO directly prevents the otherwise lethal age-related pathologies in these organs. This, in turn, could have indirect benefits for other organs. Indeed, there is some evidence that the health of the *Drosophila* gut limits lifespan (Rera et al., 2013). Furthermore, DAF-16/dFOXO could regulate key metabolic genes in these tissues, such as lipases, fatty acid catabolic genes, and others, effecting a shift in energy utilization toward prolonged health and survival. However, dFOXO activity in other tissues, such as the muscle (Demontis and Perrimon, 2010) or the mNSC (Figure 1D), can also extend lifespan. Although it is conceivable that multiple tissues independently and simultaneously limit lifespan, in at least some of these interventions, the relevant effects must be cell nonautonomous.

DAF-16 in one tissue is known to trigger DAF-16-independent responses in other tissues (Qi et al., 2012; Zhang et al., 2013). In one case, this is mediated by induction of a transcriptional mediator, *mdt-15*, and is required in part for the beneficial effects of the intestinal activation of DAF-16 by *daf-2(−)* on whole-organism aging (Zhang et al., 2013). Our results indicate that dFOXO

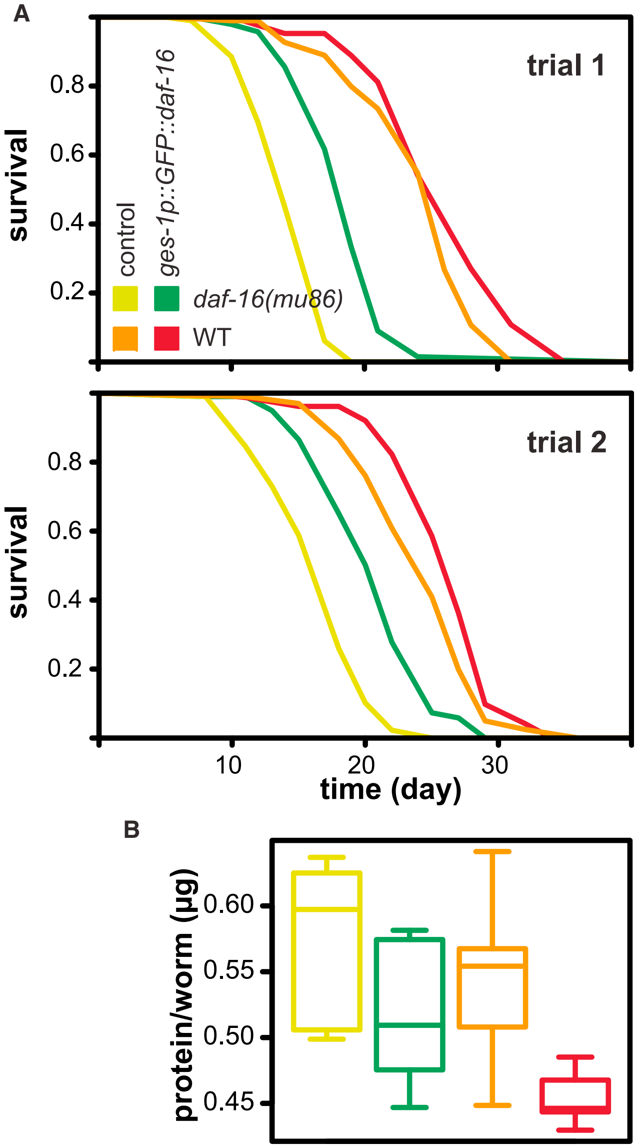

Figure 3. *daf-16*-to-*daf-16* Signaling Is Not Essential for the Lifespan Benefit of Increased DAF-16 Activity in the Intestine

(A) Survival of wild-type (WT) and *daf-16(mu86)* worms in combination with *daf-16* overexpression from a gut-specific promoter (*muEx211[ges-1p::GFP::daf-16]*) determined in two experimental trials (top and bottom panel) in worms fed HT115 bacteria.

(B) Worm protein content under the four conditions described in (A).

Color codes are the same in (A) and (B). See Figure S3A for further lifespan trials and Table 1 for statistical analysis. See also Figures S3B and S3D for the effect of an independent transgene. See Figure S3C for the lifespan effects on OP50 bacteria.

may also initiate *dfoxo*-independent processes in the receiving tissues that counteract whole-organism aging. This is the most likely mechanism whereby its activity in the *Drosophila* mNSC can extend lifespan, and a similar mechanism may underlie the health benefits observed when it is induced in the gut and fat body. The search for the factors that mediate this effect of dFOXO at a distance is now of interest, and we identified *Nplp4* as a candidate. The evolutionary persistence of this FoxO-to-other signaling between the fly and the worm strongly suggests that its relevance may extend to mammals.

EXPERIMENTAL PROCEDURES

Fly Husbandry and Experiments

All transgenes and the *dfoxo* mutant were backcrossed at least six times into the wild-type outbred Dahomey population carrying the w^{1118} mutation and cured of *Wolbachia* infection and frequently outcrossed back into the wild-type population. The Dahomey stock was collected in 1970 in Dahomey (now Benin) and has been kept in population cages maintaining its lifespan and fecundity at levels similar to freshly caught stocks. The lines were maintained, and all experiments performed, at 25°C with 60% humidity and 12 hr:12 hr light:dark cycle on sugar-yeast-agar (1SYA) food (Bass et al., 2007). Experimental flies developed at standardized densities and once-mated females were sorted on day 2 of adulthood onto food containing 200 μM RU486 (Sigma) or control food as required (15 per vial for climbing assays, five for feeding, and ten for all others). Flies were harvested on day 7 for weight and metabolite measurements and protein and RNA analysis. Sample preparation and hybridizations to Dros2 Affymetrix arrays were performed and data analyzed with LIMMA, essentially as described elsewhere (Alic et al., 2011a). For further details, see Supplemental Experimental Procedures. Gene lists are given in Table S1.

Worm Husbandry and Experiments

Worms were maintained at 20°C unless otherwise indicated. Prior to experiments, animals were maintained at the permissive temperature and grown for at least one generation in the presence of food to assure full viability. Lifespan assays were performed on HT115 bacteria carrying empty pL4440 vector, or OP50 bacteria, in the presence of 10 μM FUdR. Worms were placed on these plates at the L4 stage and scored as dead or alive every 2–3 days. For further details, see Supplemental Experimental Procedures.

Statistical Analysis

Analyses were performed in JMP (SAS) or R. Further details are given in Table 1 and Supplemental Experimental Procedures. To determine difference in slopes of the regression lines between the two gene sets in Figure 2A, the linear model was fitted with RU486-induced response in *dfoxo*Δ/Δ S_1106>*dfoxo* as the dependent variable and the response in S_1106>*dfoxo* (continuous) and gene set (categorical) as explanatory variables, testing for the significance of the interaction term.

ACCESSION NUMBERS

The ArrayExpress accession number for the array data reported in this paper is E-MTAB-1232.

SUPPLEMENTAL INFORMATION

Supplemental Information includes Supplemental Experimental Procedures, three figures, and one table and can be found with this article online at http://dx.doi.org/10.1016/j.celrep.2014.01.015.

ACKNOWLEDGMENTS

Some worm strains were provided by the Caenorhabditis Genetics Center, which is funded by National Institutes of Health Office of Research Infrastructure Programs (P40 OD010440). We acknowledge funding by the Wellcome Trust (to D.G. and L.P.) and Max Planck (to L.P.). This work is dedicated to the memory of Pedro Martinez.

Received: October 9, 2013
Revised: December 16, 2013
Accepted: January 14, 2014
Published: February 6, 2014

REFERENCES

Alic, N., Andrews, T.D., Giannakou, M.E., Papatheodorou, I., Slack, C., Hoddinott, M.P., Cochemé, H.M., Schuster, E.F., Thornton, J.M., and Partridge, L. (2011a). Genome-wide dFOXO targets and topology of the transcriptomic response to stress and insulin signalling. Mol. Syst. Biol. *7*, 502.

Alic, N., Hoddinott, M.P., Vinti, G., and Partridge, L. (2011b). Lifespan extension by increased expression of the Drosophila homologue of the IGFBP7 tumour suppressor. Aging Cell *10*, 137–147.

Bai, H., Kang, P., and Tatar, M. (2012). Drosophila insulin-like peptide-6 (dilp6) expression from fat body extends lifespan and represses secretion of Drosophila insulin-like peptide-2 from the brain. Aging Cell *11*, 978–985.

Bai, H., Kang, P., Hernandez, A.M., and Tatar, M. (2013). Activin signaling targeted by insulin/dFOXO regulates aging and muscle proteostasis in Drosophila. PLoS Genet. *9*, e1003941.

Bass, T.M., Grandison, R.C., Wong, R., Martinez, P., Partridge, L., and Piper, M.D. (2007). Optimization of dietary restriction protocols in Drosophila. J. Gerontol. A Biol. Sci. Med. Sci. *62*, 1071–1081.

Broughton, S.J., Piper, M.D., Ikeya, T., Bass, T.M., Jacobson, J., Driege, Y., Martinez, P., Hafen, E., Withers, D.J., Leevers, S.J., and Partridge, L. (2005). Longer lifespan, altered metabolism, and stress resistance in Drosophila from ablation of cells making insulin-like ligands. Proc. Natl. Acad. Sci. USA *102*, 3105–3110.

Brunet, A., Bonni, A., Zigmond, M.J., Lin, M.Z., Juo, P., Hu, L.S., Anderson, M.J., Arden, K.C., Blenis, J., and Greenberg, M.E. (1999). Akt promotes cell survival by phosphorylating and inhibiting a Forkhead transcription factor. Cell *96*, 857–868.

Cook-Wiens, E., and Grotewiel, M.S. (2002). Dissociation between functional senescence and oxidative stress resistance in Drosophila. Exp. Gerontol. *37*, 1347–1357.

Demontis, F., and Perrimon, N. (2010). FOXO/4E-BP signaling in Drosophila muscles regulates organism-wide proteostasis during aging. Cell *143*, 813–825.

Flachsbart, F., Caliebe, A., Kleindorp, R., Blanché, H., von Eller-Eberstein, H., Nikolaus, S., Schreiber, S., and Nebel, A. (2009). Association of FOXO3A variation with human longevity confirmed in German centenarians. Proc. Natl. Acad. Sci. USA *106*, 2700–2705.

Giannakou, M.E., Goss, M., Jünger, M.A., Hafen, E., Leevers, S.J., and Partridge, L. (2004). Long-lived Drosophila with overexpressed dFOXO in adult fat body. Science *305*, 361.

Giannakou, M.E., Goss, M., and Partridge, L. (2008). Role of dFOXO in lifespan extension by dietary restriction in Drosophila melanogaster: not required, but its activity modulates the response. Aging Cell *7*, 187–198.

Hwangbo, D.S., Gershman, B., Tu, M.P., Palmer, M., and Tatar, M. (2004). Drosophila dFOXO controls lifespan and regulates insulin signalling in brain and fat body. Nature *429*, 562–566.

Kenyon, C., Chang, J., Gensch, E., Rudner, A., and Tabtiang, R. (1993). A C. elegans mutant that lives twice as long as wild type. Nature *366*, 461–464.

Kuningas, M., Mägi, R., Westendorp, R.G., Slagboom, P.E., Remm, M., and van Heemst, D. (2007). Haplotypes in the human Foxo1a and Foxo3a genes; impact on disease and mortality at old age. Eur. J. Hum. Genet. *15*, 294–301.

Libina, N., Berman, J.R., and Kenyon, C. (2003). Tissue-specific activities of C. elegans DAF-16 in the regulation of lifespan. Cell *115*, 489–502.

Maier, W., Adilov, B., Regenass, M., and Alcedo, J. (2010). A neuromedin U receptor acts with the sensory system to modulate food type-dependent effects on C. elegans lifespan. PLoS Biol. *8*, e1000376.

Min, K.J., Yamamoto, R., Buch, S., Pankratz, M., and Tatar, M. (2008). Drosophila lifespan control by dietary restriction independent of insulin-like signaling. Aging Cell *7*, 199–206.

Murphy, C.T., Lee, S.J., and Kenyon, C. (2007). Tissue entrainment by feedback regulation of insulin gene expression in the endoderm of Caenorhabditis elegans. Proc. Natl. Acad. Sci. USA *104*, 19046–19050.

Nässel, D.R., and Winther, A.M. (2010). Drosophila neuropeptides in regulation of physiology and behavior. Prog. Neurobiol. *92*, 42–104.

Partridge, L., and Brüning, J.C. (2008). Forkhead transcription factors and ageing. Oncogene *27*, 2351–2363.

Poirier, L., Shane, A., Zheng, J., and Seroude, L. (2008). Characterization of the Drosophila gene-switch system in aging studies: a cautionary tale. Aging Cell *7*, 758–770.

Postnikoff, S.D., Malo, M.E., Wong, B., and Harkness, T.A. (2012). The yeast forkhead transcription factors fkh1 and fkh2 regulate lifespan and stress response together with the anaphase-promoting complex. PLoS Genet. *8*, e1002583.

Qi, W., Huang, X., Neumann-Haefelin, E., Schulze, E., and Baumeister, R. (2012). Cell-nonautonomous signaling of FOXO/DAF-16 to the stem cells of Caenorhabditis elegans. PLoS Genet. *8*, e1002836.

Rera, M., Azizi, M.J., and Walker, D.W. (2013). Organ-specific mediation of lifespan extension: more than a gut feeling? Ageing Res. Rev. *12*, 436–444.

Salih, D.A., and Brunet, A. (2008). FoxO transcription factors in the maintenance of cellular homeostasis during aging. Curr. Opin. Cell Biol. *20*, 126–136.

Slack, C., Giannakou, M.E., Foley, A., Goss, M., and Partridge, L. (2011). dFOXO-independent effects of reduced insulin-like signaling in Drosophila. Aging Cell *10*, 735–748.

Willcox, B.J., Donlon, T.A., He, Q., Chen, R., Grove, J.S., Yano, K., Masaki, K.H., Willcox, D.C., Rodriguez, B., and Curb, J.D. (2008). FOXO3A genotype is strongly associated with human longevity. Proc. Natl. Acad. Sci. USA *105*, 13987–13992.

Wong, R., Piper, M.D., Wertheim, B., and Partridge, L. (2009). Quantification of food intake in Drosophila. PLoS ONE *4*, e6063.

Yamamoto, R., and Tatar, M. (2011). Insulin receptor substrate chico acts with the transcription factor FOXO to extend Drosophila lifespan. Aging Cell *10*, 729–732.

Zhang, P., Judy, M., Lee, S.J., and Kenyon, C. (2013). Direct and indirect gene regulation by a life-extending FOXO protein in C. elegans: roles for GATA factors and lipid gene regulators. Cell Metab. *17*, 85–100.

Cell Reports

Supplemental Information

Cell-Nonautonomous Effects of dFOXO/DAF-16 in Aging

Nazif Alic, Jennifer M. Tullet, Teresa Niccoli, Susan Broughton, Matthew P. Hoddinott, Cathy Slack,

David Gems, and Linda Partridge

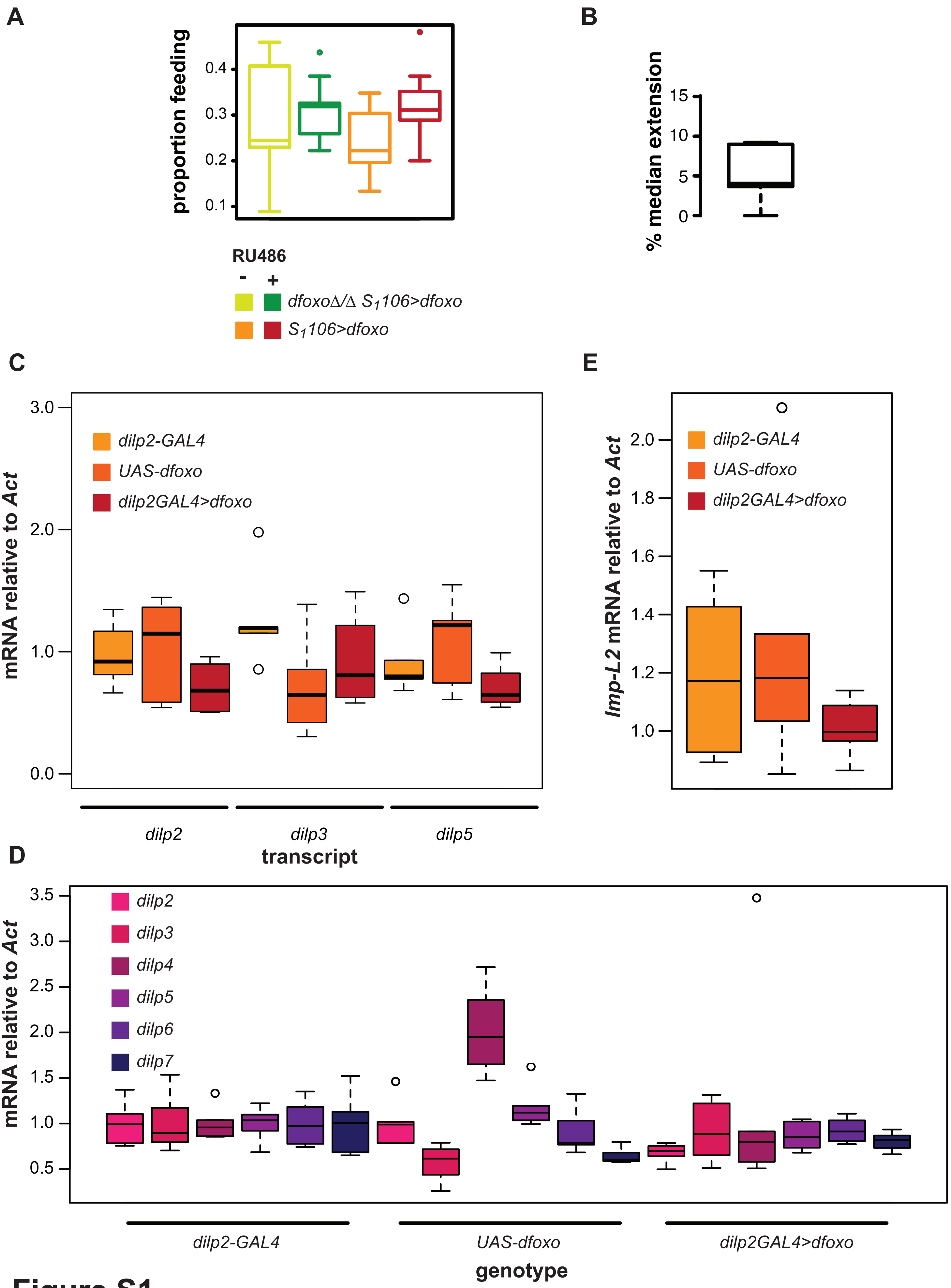
A
proportion feeding
0.4
0.3
0.2
0.1
RU486
-
+
dfoxoΔ/Δ S1106>dfoxo
S1106>dfoxo
B
% median extension
15
10
5
0
C
3.0
2.0
1.0
0.0
mRNA relative to Act
dilp2-GAL4
UAS-dfoxo
dilp2GAL4>dfoxo
dilp2
dilp3
dilp5
transcript
E
Imp-L2 mRNA relative to Act
2.0
1.8
1.6
1.4
1.2
1.0
dilp2-GAL4
UAS-dfoxo
dilp2GAL4>dfoxo
D
mRNA relative to Act
3.5
3.0
2.5
2.0
1.5
1.0
0.5
dilp2
dilp3
dilp4
dilp5
dilp6
dilp7
dilp2-GAL4
UAS-dfoxo
dilp2GAL4>dfoxo
genotype

Figure S1

Figure S1

A Feeding events as proportion of total observed events (fly occurrences) per vial for the *$S_1$106>dfoxo* and *dfoxoΔ/Δ $S_1$106>dfoxo* females (15 and 9 vials per food condition, respectively) on food with or without RU486. The data were analysed with a Generalised Linear Model with binomial distribution and an overdispersion parameter. There was no significant effect of genotype (p=0.4), presence of RU486 (p=0.08) or their interaction (p=0.4). **B** Box plot representing the median lifespan extension (as % of control) caused by RU486 feeding in *$S_1$106>dfoxo* females in six independent trials. Log-rank test revealed that RU486 had a significant effect in each case (p<0.05). **C** mRNA levels of *dilp2*, *dilp3* and *dilp5* in heads of female flies of the indicated genotype. Data were analysed with a Linear Model and no significant effect of transcript or genotype, or any interaction, was found (p>0.05, n=4-5). **D** mRNA levels of *dilp2* through to *dilp7* in whole female flies of the indicated genotype, with the level in *dilp2GAL4* control set to 1 and n=6. Data were analysed with a Linear Model and no significant effect of genotype was found (p=0.2), while there was a significant effect of the transcript ($p<10^{-4}$) and a significant interaction of the two ($p=2x10^{-4}$), but in no case was *dilp2GAL4>dfoxo* significantly different to both of the controls by t-test (p>0.05). Note the levels of *dilp4* in the *UAS-dfoxo* control are different to *dilp4* levels in the two other genotypes, and this is most likely due to the effect of the driver alone on *dilp4* levels. *dilp1* transcript is undetectable in whole adult females. **E** mRNA levels of *Imp-L2* in whole female flies of the indicated genotype, with the level in *dilp2GAL4* control set to 1 and n=6. ANOVA revealed no significant differences (p=0.3).

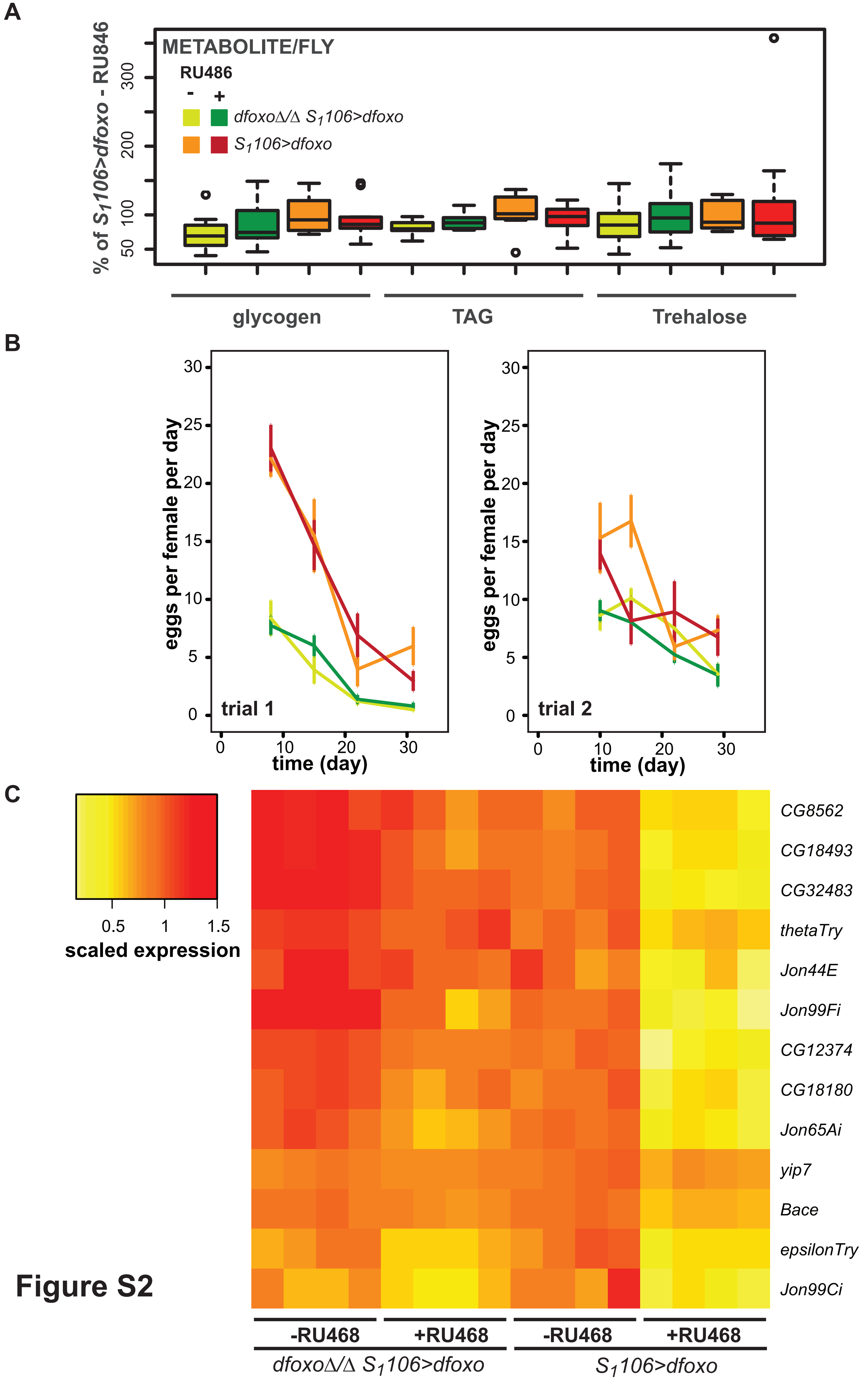
A
METABOLITE/FLY
RU486
- +
dfoxoΔ/Δ S1106>dfoxo
S1106>dfoxo
% of S1106>dfoxo - RU846
50
100
200
300
glycogen
TAG
Trehalose
B
eggs per female per day
0
5
10
15
20
25
30
trial 1
trial 2
time (day)
0
10
20
30
C
0.5
1
1.5
scaled expression
CG8562
CG18493
CG32483
thetaTry
Jon44E
Jon99Fi
CG12374
CG18180
Jon65Ai
yip7
Bace
epsilonTry
Jon99Ci
-RU468
+RU468
-RU468
+RU468
dfoxoΔ/Δ S1106>dfoxo
S1106>dfoxo

Figure S2

Figure S2

A Trehalose, TAG and glycogen were quantified per fly for the four conditions shown (n=8-15 per condition). Data were analysed per compound with a Mixed Effect Linear Model with genotype and RU486 as main effects, their interaction, and experimental batch as random effect. Only the effect of genotype for glycogen was borderline significant (p=0.050), all others were not. **B** Eggs laid per female per 24h for the same four conditions over the first ~4 weeks of life determined in two experimental trials (two panels, means ± SEM). The data were analysed with Mixed Effect Linear Model with time, genotype and RU486 as main effects, their interactions, and experimental trial as random effect. The effects of genotype (genomic *dfoxo*), time and their interaction were all significant ($p<10^{-4}$) but RU486 or any of its interactions were not (p>0.05). The same colour code is used in A and B. **C** Heatmap of the expression levels detected by microarray analysis for proteolysis genes that show a different response to RU486 in *$S_1$106>dfoxo* and *dfoxo*Δ/Δ *$S_1$106>dfoxo* females. Each column represents a separate array with the expression levels scaled to the average of the *$S_1$106>dfoxo* -RU486 condition.

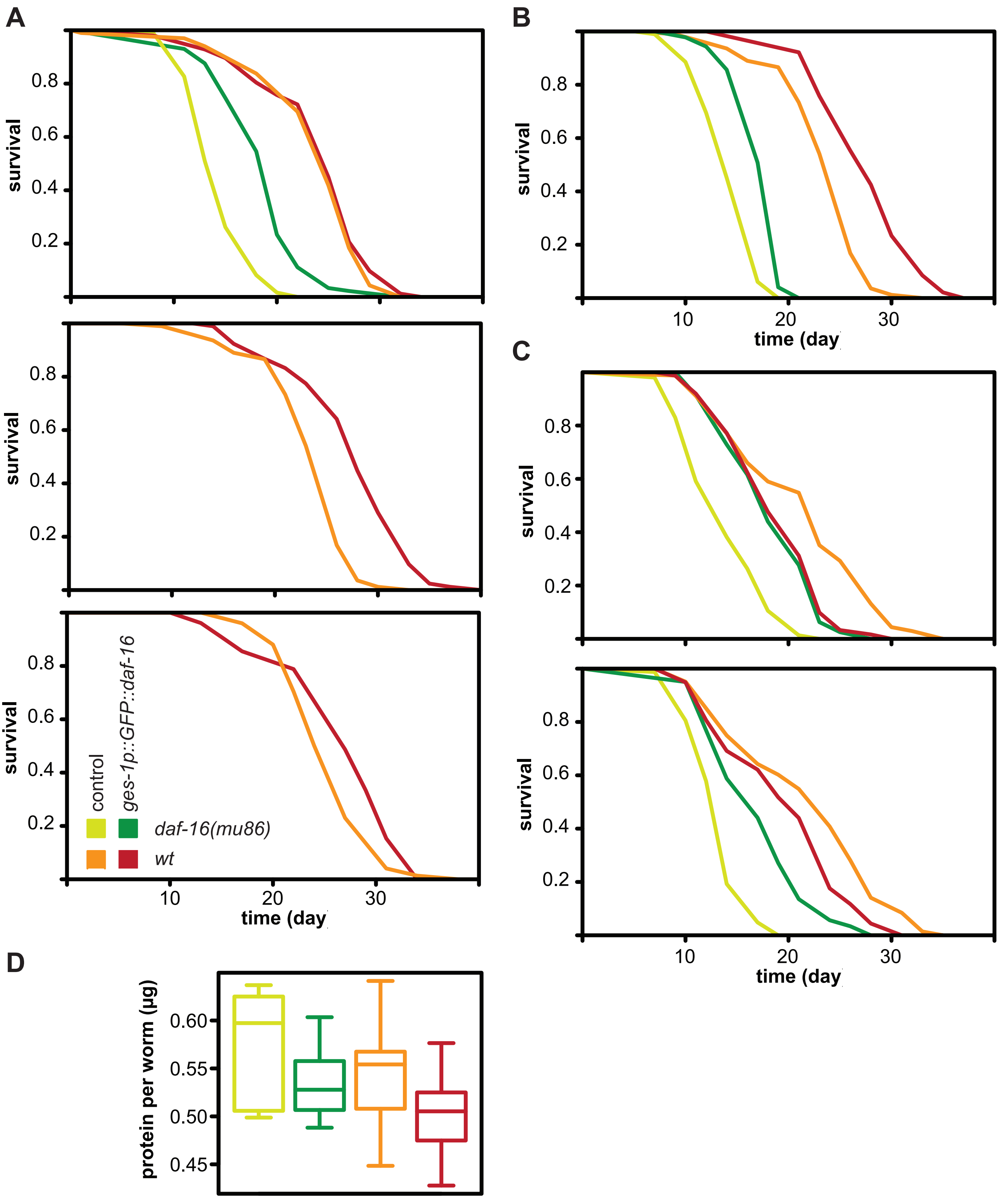
A
B
C
D
survival
0.8
0.6
0.4
0.2
10
20
30
time (day
control
ges-1p::GFP::daf-16
daf-16(mu86)
wt
protein per worm (µg)
0.60
0.55
0.50
0.45

Figure S3

Figure S3

A Further experimental trials looking at the effect of gut over-expression of *daf-16* (*muEx211[ges-1p::GFP::daf-16]*) in wild-type and *daf-16* deficient worms on HT115 bacteria. Each panel is a separate experimental trial. **B** The effect of an independently derived transgene (*muEx227[ges-1p::GFP::daf-16]*) on lifespan in wild-type and *daf-16* deficient worms on HT115 bacteria. Log-rank test detected significant differences in both wild-type and *daf-16* deficient worms ($p<10^{-4}$). **C** Two experimental trials looking at the effect of gut over-expression of *daf-16* (*muEx211[ges-1p::GFP::daf-16]*) in wild-type and *daf-16* deficient worms on OP50 bacteria. Log-rank test detected significant differences in both wild-type and *daf-16* deficient worms ($p<0.01$ and $p<10^{-4}$, respectively) in both trials. MECPH model revealed significant interaction between genomic *daf-16* and its intestinal induction ($p<10^{-15}$). **D** Protein content of worms with *daf-16* induced in the intestine (*muEx227[ges-1p::GFP::daf-16]*) in otherwise daf-16(+) or *daf-16(mu86)* worms. The data were analysed with a Linear model and the effect of transgene found significant (p=0.012), the effect of daf-16 mutation borderline significant (p=0.069) and no significant interaction (p=0.86, n=8-16). The colour code is the same in all panels and is given in A.

Table S1 Supplemental Data

Separate Excel File containing:

- List of genes differentially expressed (10% FRD) in S106>dfoxo females with RU486 treatment, with indication of the ones that show significant interaction between genotype (S106>dfoxo or dfoxo null S106>dfoxo) and RU486 (10% FDR).
- Biological process categories over-represented in genes with significant interaction term in list above.

Extended Experimental Procedures

Fly husbandry, lifespan, feeding and climbing assays

$S_1$106 (Giannakou et al., 2004; Poirier et al., 2008), *UAS-dfoxo* (Giannakou et al., 2004), *dilp2-GAL4* (Ikeya et al., 2002) and *dfoxo*Δ^{94} (Slack et al., 2011) were backcrossed at least 6 times into the wild-type outbred Dahomey population carrying the w^{1118} mutation and cured of *Wolbachia* infection, and frequently outcrossed back into the wild-type population. The Dahomey stock was collected in 1970 in Dahomey (now Benin) and has been kept in population cages maintaining its lifespan and fecundity at levels similar to freshly caught stocks. Combinations of transgenes/mutants were created using standard fly genetic techniques while avoiding population bottlenecks. *dfoxo*Δ^{94} allele was tracked with PCR (Slack et al., 2011). The lines were maintained, and all experiments performed, at 25ºC with 60% humidity and 12h:12h light:dark cycle, on sugar-yeast-agar (1SYA) food (Bass et al., 2007). Experimental flies developed at standardised densities and once-mated females were sorted on day two of adulthood onto food containing 200 μM RU486 (Sigma) or control food as required (15 per vial for climbing assays, 5 for feeding, 10 for all others). Note that the food from the exact same cook was used for the - and + RU486. Lifespan measurements were performed essentially as described (Giannakou et al., 2004). Climbing ability was scored once a week. Flies were placed in a plastic pipette (15 flies per pipette, 3 pipettes per condition), tapped to the bottom and allowed to climb for 45 seconds. Their position was then scored as low (climbing below 2 cm), medium (above 2 and below 20) and high (above 20 cm). This scoring was repeated 3 times for each pipette and averaged to the nearest fly. Feeding

observations were performed essentially as described (Wong et al., 2009) for one hour on days 7 and 8 by two observers, blinded to the experimental conditions, with flies feeding (proboscis extended to the food) or not scored multiple times for each vial. The observations were summed per vial before analysis. Flies were harvested on day seven for weight and metabolite measurement, protein and RNA analysis, by freezing in liquid N_2.

Metabolite and weight measurements

Whole fly trehalose was determined as described (Alic et al., 2011b). For triacylglycerol (TAG) and protein measurements, a single female was homogenised in 400 µl of 0.05% Tween-20 and TAG measured with Infinity TAG Reagent (Thermo Scientific), protein with BCA Protein Assay kit (Pierce) against a suitable standard. For glycogen measurements, a single female was homogenised into 100 µl of PBS 0.1% TritonX-100, and the glucose released by incubation with amyloglucosidase in the samples and glycogen standards determined with Infinity Glucose Reagent (Thermo Scientific). Flies were weighted singly or in pairs, and the two highest/lowest measurements for each condition discarded.

Western blots, qPCR and microarray analysis

Proteins were extracted and western blots performed as described (Alic et al., 2011b). RNA extractions and qPCR were performed essentially as described (Alic et al., 2011a). *dfoxo* primers used were: GTTGGCCCCAGGAGACTC and GATGAGATCCGCATAGGATAG; *Actin5C*: CACACCAAATCTTACAAAATGTGTGA and AATCCGGCCTTGCACATG; *Nplp4*: CCGGACCATGCATTGCAATG and GCGCATATCCGTAATCCGT. *dfoxo* mRNA was deemed detectable when the qPCR signal was above that

observed in the absence of reverse transcriptase. mRNA levels of *dilp2* through to 7 and *Imp-L2* were quantified as described (Alic et al., 2011b; Broughton et al., 2005; Gronke et al., 2010). Sample preparation and hybridisations to Dros2 Affymetrix arrays were performed as described (Alic et al., 2011a). The microarray data were analysed in R. They were summarised and normalised using RMA, differential expression was assessed using Linear Models and the empirical Bayes moderated t-statistic implemented in LIMMA (Bolstad et al., 2003; Irizarry et al., 2003a; Irizarry et al., 2003b) and FDR was controlled using the described procedure (Benjamini and Hochberg, 1995). Genes differentially expressed on RU486 feeding in *$S_1$106>dfoxo* females were determined (at FDR=10%), and amongst these genes the ones with significant interaction between genotype and RU486 (FDR=10%) were determined by a Linear Model (full factorial design) fitted to all the data (the four conditions). Gene Ontology Enrichment was determined with David EASE (Dennis et al., 2003). Gene lists are given in **Supplemental Data**. Raw microarray data are available from ArrayExpress under accession code: E-MTAB-1232.

Worm husbandry, lifespan and protein assays

Worms were maintained at 20°C unless otherwise indicated. The following strains were used: N2s derived from the Caenorhabditis Genetics Center male N2 line, XA2954 *daf-16(mu86)* derived from CF1038 *daf-16(mu86)* (Lin et al., 2001), GA1064 *muEx211[ges-1p::GFP::daf-16]*, GA1065 *daf-16(mu86) muEx211[ges-1p::GFP::daf-16]*, GA1067 *muEx227[ges-1p::GFP::daf-16]*, GA1066 *daf-16(mu86) muEx227[ges-1p::GFP::daf-16]*. The latter four strains were created by crossing CF1514 *daf-16(mu86); daf-*

2(e1370) muEx211[ges-1p::GFP::daf-16] or CF1595 *daf-16(mu86); daf-2(e1370) muEx227[ges-1p::GFP::daf-16]* (Libina et al., 2003) with the N2 strain and isolating wild type and *daf-16* mutant worms carrying the extrachromosomal array using three-primer PCR: F: CGGTGACCATCTAGAGTCACA; In: CCAATAGCTGGAGAAACACGA and; R: CTAGGAGGAAAAGCCATTTGT (Love et al., 2010), without further outcrossing. Prior to experiments animals were maintained at the permissive temperature and grown for at least one generation in the presence of food to assure full viability. Lifespan assays were performed on HT115 bacteria carrying empty pL4440 vector, or OP50 bacteria, in the presence of 10μM FUdR. Worms were placed on these plates at the L4 stage and scored as dead or alive every 2-3 days. For protein measurements, 20 L4s were picked from OP50 maintenance plates and allowed to mature for 24h on HTT15 bacteria carrying empty pL4440 vector, picked into 100 μl of 0.05% Tween-20, the bacteria removed by centrifugation and the worms lysed with glass beads in a Ribolyser. Protein concentration was determined against a BSA standard as for the fly. 10-20 repeats were performed for each sample and 5% lowest and 5% highest measurements removed from each group.

Statistical Analysis

Mixed Effect Linear Models, Linear Models, Generalised Linear Models, post-hoc tests and Log-rank comparisons were performed in JMP (SAS), all other analysis in R. MECPH analysis was performed using the *coxme* package (Therneau, T., 2012, http://CRAN.R-project.org/package=coxme), CPH using *survival* (Therneau, T., 2013, http://CRAN.R-project.org/package=survival), Mixed Effects Ordinal Logistic analysis using

ordinal (Christensen, R. H. B., 2012 http://www.cran.r-project.org/package=ordinal). Further details are given in **Table 1**. To determine difference in slopes of the regression lines between the two gene sets in Figure 2A, Linear Model was fitted with RU486-induced response in *dfoxo*Δ/Δ *$S_1$106>dfoxo* as the dependant variable and the response in *$S_1$106>dfoxo* (continuous) and gene set (categorical) as explanatory variables, testing for the significance of the interaction term.

Supplemental References

Benjamini, Y., and Hochberg, Y. (1995). Controlling the False Discovery Rate: A practical and Powerful Approach to Multiple Testing. J. R. Stat. Soc. Ser. B Stat. Methodol. 57, 289-300.

Bolstad, B.M., Irizarry, R.A., Astrand, M., and Speed, T.P. (2003). A comparison of normalization methods for high density oligonucleotide array data based on variance and bias. Bioinformatics 19, 185-193.

Dennis, G., Jr., Sherman, B.T., Hosack, D.A., Yang, J., Gao, W., Lane, H.C., and Lempicki, R.A. (2003). DAVID: Database for Annotation, Visualization, and Integrated Discovery. Genome Biol 4, P3.

Gronke, S., Clarke, D.-F., Broughton, S., Andrews, T.D., and Partridge, L. (2010). Molecular evolution and functional characterisation of Drosophila insulin-like peptides. PLoS Genet 6, e1000857.

Ikeya, T., Galic, M., Belawat, P., Nairz, K., and Hafen, E. (2002). Nutrient-dependent expression of insulin-like peptides from neuroendocrine cells in the CNS contributes to growth regulation in Drosophila. Curr Biol 12, 1293-1300.

Irizarry, R.A., Bolstad, B.M., Collin, F., Cope, L.M., Hobbs, B., and Speed, T.P. (2003a). Summaries of Affymetrix GeneChip probe level data. Nucleic Acids Res 31, e15.

Irizarry, R.A., Hobbs, B., Collin, F., Beazer-Barclay, Y.D., Antonellis, K.J., Scherf, U., and Speed, T.P. (2003b). Exploration, normalization, and summaries of high density oligonucleotide array probe level data. Biostatistics 4, 249-264.

Lin, K., Hsin, H., Libina, N., and Kenyon, C. (2001). Regulation of the Caenorhabditis elegans longevity protein DAF-16 by insulin/IGF-1 and germline signaling. Nat Genet 28, 139-145.

Love, D.C., Ghosh, S., Mondoux, M.A., Fukushige, T., Wang, P., Wilson, M.A., Iser, W.B., Wolkow, C.A., Krause, M.W., and Hanover, J.A. (2010). Dynamic O-GlcNAc cycling at promoters of Caenorhabditis elegans genes regulating longevity, stress, and immunity. Proc Natl Acad Sci U S A 107, 7413-7418.
